# Reversible switching of arylazopyrazole within a metal–organic cage

**DOI:** 10.3762/bjoc.15.232

**Published:** 2019-10-10

**Authors:** Anton I Hanopolskyi, Soumen De, Michał J Białek, Yael Diskin-Posner, Liat Avram, Moran Feller, Rafal Klajn

**Affiliations:** 1 Department of Organic Chemistry, Weizmann Institute of Science, Rehovot 76100, Israel; 2 Chemical Research Support, Weizmann Institute of Science, Rehovot 76100, Israel

**Keywords:** arylazopyrazoles, coordination cages, inclusion complexes, molecular switches, photochromism

## Abstract

Arylazopyrazoles represent a new family of molecular photoswitches characterized by a near-quantitative conversion between two states and long thermal half-lives of the metastable state. Here, we investigated the behavior of a model arylazopyrazole in the presence of a self-assembled cage based on Pd–imidazole coordination. Owing to its high water solubility, the cage can solubilize the *E* isomer of arylazopyrazole, which, by itself, is not soluble in water. NMR spectroscopy and X-ray crystallography have independently demonstrated that each cage can encapsulate two molecules of *E*-arylazopyrazole. UV-induced switching to the *Z* isomer was accompanied by the release of one of the two guests from the cage and the formation of a 1:1 cage/*Z*-arylazopyrazole inclusion complex. DFT calculations suggest that this process involves a dramatic change in the conformation of the cage. Back-isomerization was induced with green light and resulted in the initial 1:2 cage/*E*-arylazopyrazole complex. This back-isomerization reaction also proceeded in the dark, with a rate significantly higher than in the absence of the cage.

## Introduction

The importance of confinement for chemical reactivity is becoming increasingly appreciated. Confining reactive species to small volumes can greatly increase their effective molarity and consequently, promote chemical reactions. Such reaction acceleration has been demonstrated at interfaces [1–3], in self-assembled cages [4–7], on DNA chains [8], and on the surfaces of inorganic nanoparticles [9–10]. Additionally, nanosized cages can induce preorganization of the encapsulated species, thus inducing unusual regioselectivities in various chemical reactions [11–13]. The limited dimensions of cage cavities can also be used for arresting the growth of polycondensates at an early stage, thereby making it possible to isolate labile species. For example, Fujita and co-workers demonstrated the stabilization of cyclic trimers of siloxanes inside small self-assembled cages [14] and, using larger cages, the synthesis of 4 nm silica nanoparticles, which would be difficult to obtain otherwise [15].

One class of organic compounds that exhibit various intriguing properties under confinement is photochromic molecules. In a seminal contribution, unusual switching patterns have been revealed within densely packed monolayers of azobenzene on planar gold [16]. In contrast, placing azobenzene inside the cavity of an octahedral cage renders it photochemically inert, stabilizing the typically metastable *Z* isomer [17]. Similarly, a related cage has recently been shown to induce negative photochromism in the spiropyran switch [18]. Other recent studies of photochromic systems within macrocyclic and supramolecular hosts [19] include dihydroazulene switches [20] and red-shifted azobenzenes [21–22] inside cucurbiturils and cyclodextrins. The behavior of light-responsive compounds can also be affected by their confinement to the surfaces of inorganic nanoparticles. Among other examples, the isomerization kinetics of azobenzene could be tuned by a factor of >5000 by the molecules with which it was co-adsorbed on gold nanoparticles [23], and donor–acceptor Stenhouse adduct (DASA) switches were found to be stabilized in their zwitterionic form when confined to iron oxide nanoparticles [24]. Similarly, the ability of anthracenes to photodimerize greatly depends on the curvature of their “host” nanoparticle [25]. Despite these advances, we are still far from achieving the ease and elegance, with which natural systems employ confinement effects to control photoisomerization reactions [26].

Recently, much effort has been devoted to developing new families of heterocyclic azo switches [27–31], largely driven by the rapid growth of the emerging field of photopharmacology [32–34]. Among them, arylazopyrazoles have attracted considerable interest because of their synthetic availability and desirable photochemical properties, such as the high thermal stability of the *Z* isomer and a large band separation between the *E* and *Z* isomers, allowing each to be addressed with a high selectivity [35–40]. Consequently, arylazopyrazoles have been employed as photoresponsive gelators [41] and adhesives [42] and for controlling antimicrobial response [43–44], cell adhesion to surfaces [45], as well as DNA [46] and microtubule [47] self-assembly using light.

**Scheme 1:**
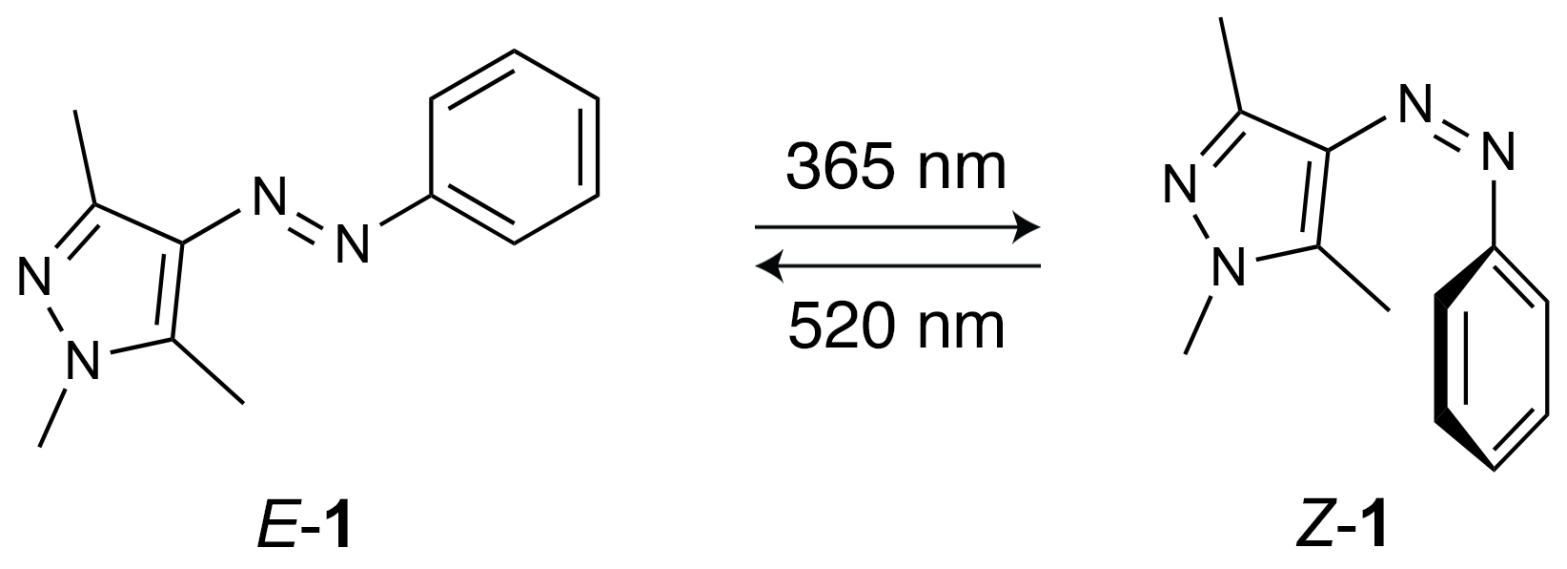
Reversible photoisomerization of phenylazotrimethylpyrazole **1**.

**Figure 1:**
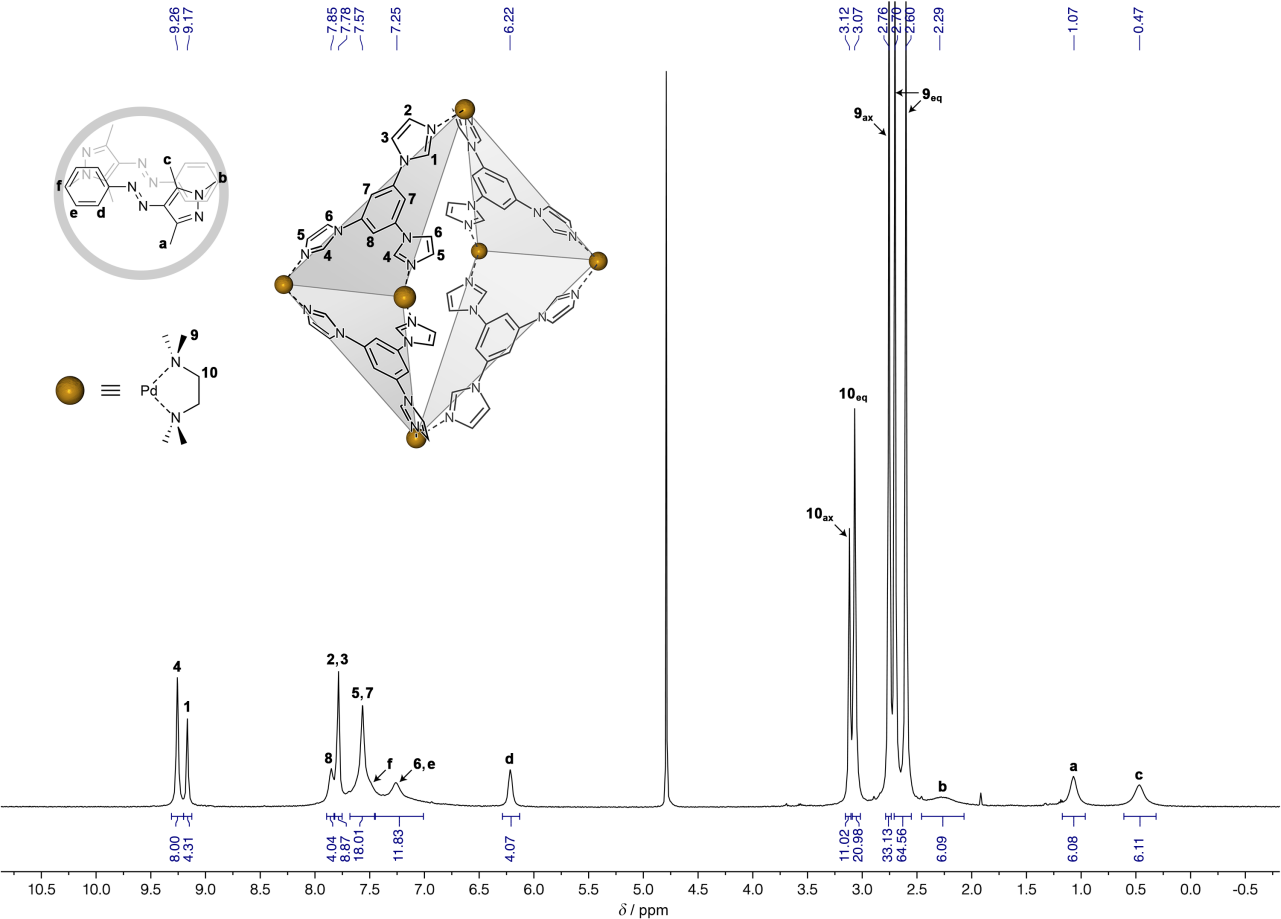
^1^H NMR spectrum of (*E*-**1**)_2_
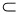
**2** (500 MHz, D_2_O, 298 K).

Here, we focused on the prototypical arylazopyrazole **1** [35] (Scheme 1) and a previously reported [48] metal–organic cage **2** (see Figure 1). We have recently demonstrated that various azobenzenes formed 2:1 inclusion complexes with **2** [49] and hypothesized, based on the structural similarity between azobenzenes and arylazopyrazoles, that **2** would similarly encapsulate *E*-**1**.

## Results and Discussion

Arylazopyrazole *E*-**1** has a very low solubility in water and its encapsulation in cage **2** was attempted from the solid state. Encouragingly, when a colorless aqueous solution of **2** was stirred with an excess of solid *E*-**1**, it turned to intense yellow within several hours. Figure 1 shows a ^1^H NMR spectrum of the resulting solution, where the identity of all the proton resonances was assigned using a combination of two-dimensional NMR techniques, including ^1^H-^1^H homonuclear correlation spectroscopy (COSY), ^1^H-^1^H nuclear Overhauser effect spectroscopy (NOESY), ^1^H-^13^C heteronuclear single quantum correlation spectroscopy (HSQC), and ^1^H-^13^C heteronuclear multiple bond correlation spectroscopy (HMBC) (see the Supporting Information File 1, Figures S5–S13). The spectrum showed the presence of all of *E*-**1**’s protons, with the resonances associated with the *ortho* protons of **1**’s phenyl ring, as well as the protons of all three methyl groups markedly upfield-shifted compared to free *E*-**1** in CDCl_3_ (see Figure S14 in Supporting Information File 1). These shifts (by ~1.0–2.2 ppm) cannot be accounted for by replacing the solvent from CDCl_3_ to D_2_O; rather, they suggest that **1** resides in the hydrophobic cavity of **2**, interacting with its aromatic walls. Integration of the signals due to **1** and **2** confirmed that each molecule of the host encapsulates two molecules of the guest.

**Figure 2:**
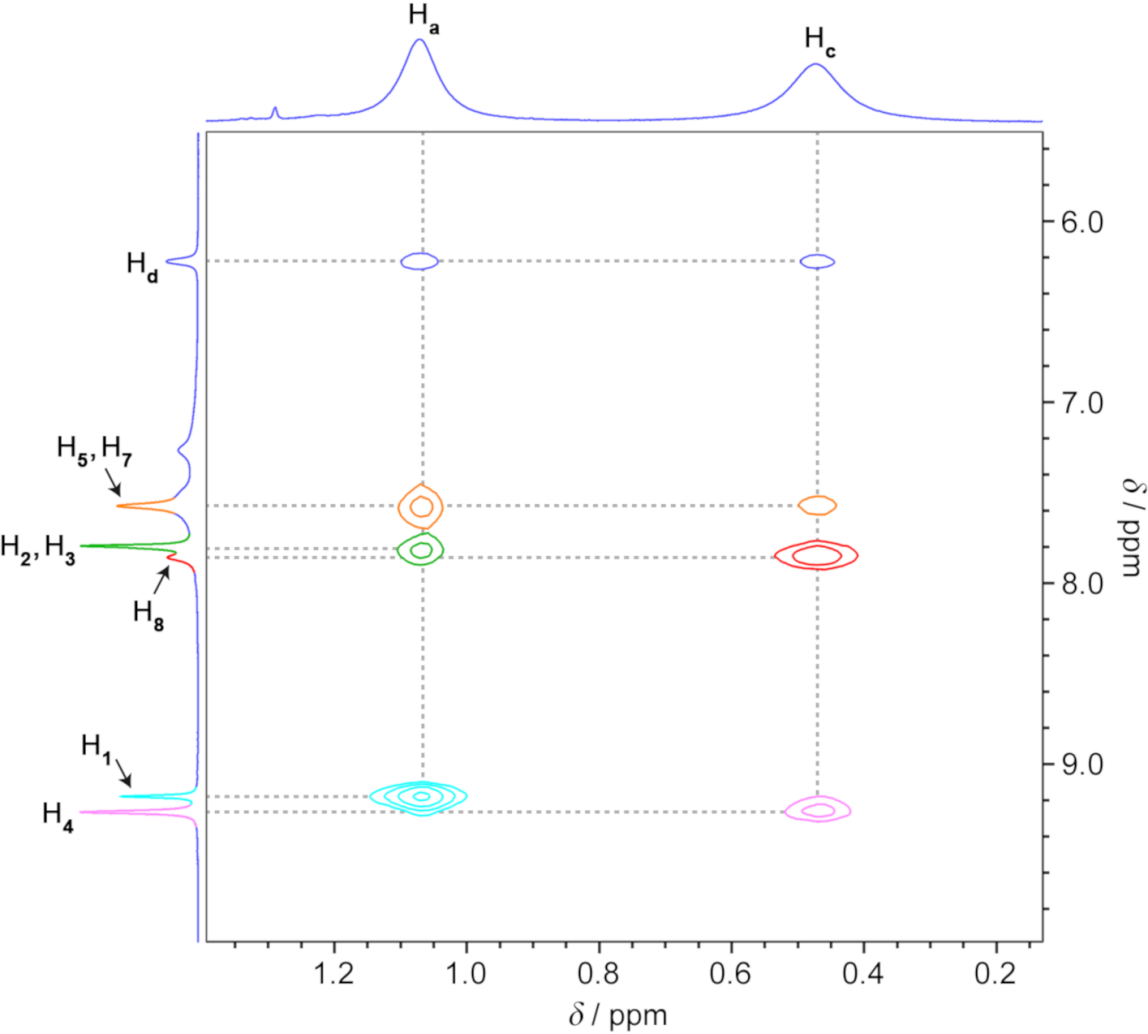
Partial ^1^H-^1^H NOESY NMR spectrum of (*E*-**1**)_2_
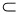
**2** showing NOE correlations between host **2** and guest **1** (500 MHz, D_2_O, 298 K) (the corresponding full-range spectrum is shown in Figure S6, Supporting Information File 1).

The presence of a complex is also evident from the NOE spectrum, which shows multiple host–guest correlations (see Figure S6 in Supporting Information File 1 for a full-range spectrum). For example, *E*-**1**’s protons H**_d_** at 6.22 ppm correlate both with the resonances at 7.78 ppm and at 7.57 ppm, which all originate from **2**’s protons (H**_2_** + H**_3_** and H**_5_** + H**_7_**, respectively). Similarly, the H**_a_** protons are correlated with H**_1_**, H**_3_**, and H**_7_**, whereas the H**_c_** protons are correlated with H**_4_**, H**_8_**, and H**_5_** (see the partial NOE spectrum in Figure 2; see below for further analysis of this spectrum).

**Figure 3:**
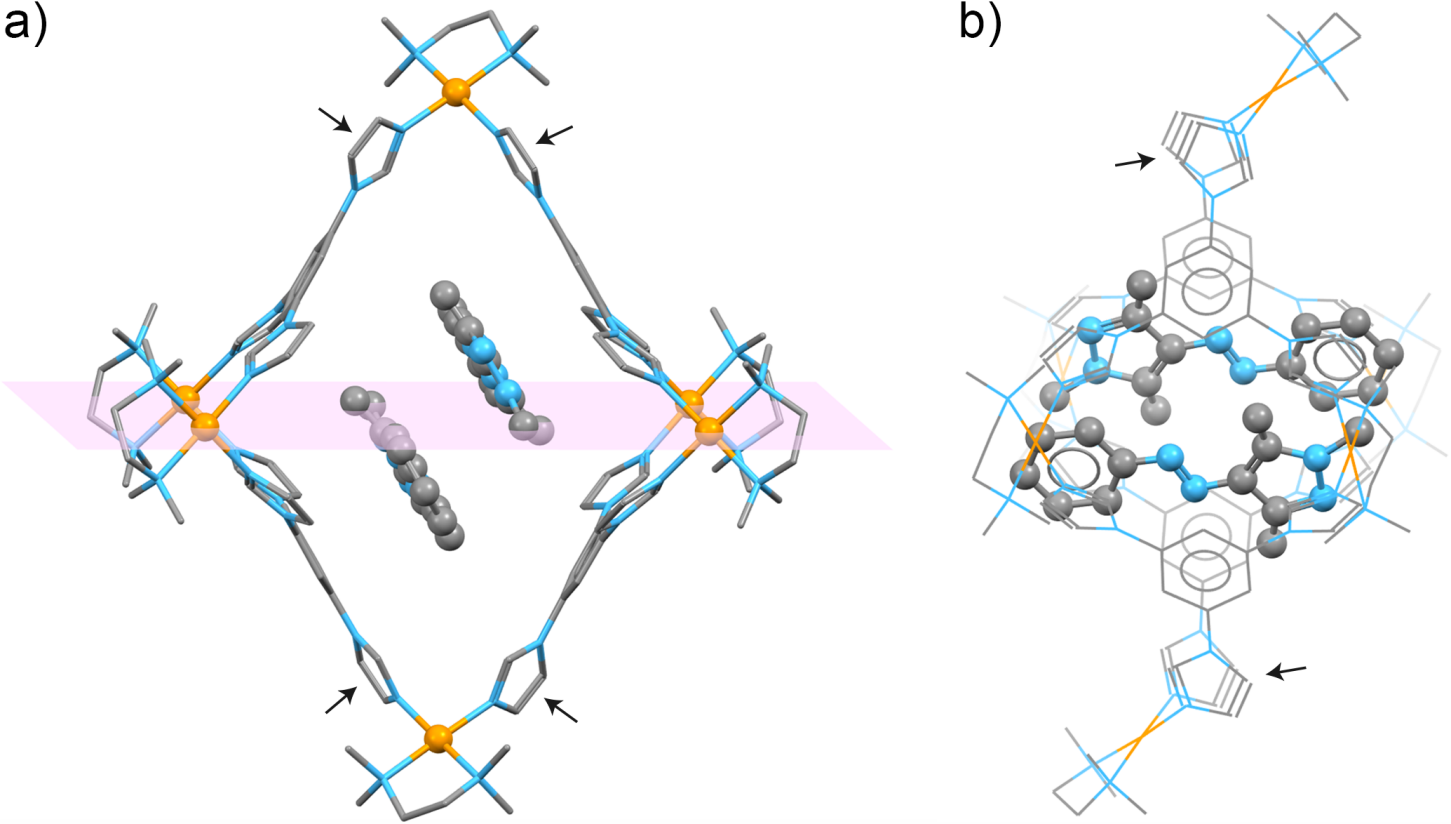
Front view (a) and side view (b) of the X-ray crystal structure of (*E*-**1**)_2_
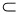
**2** (major conformations of **1** and **2**; see also Supporting Information File 1, Figures S15 and S16). The horizontal plane (pink) denotes the positions of eight equatorial imidazole groups; four axial imidazoles are denoted by black arrows. Color codes: C, gray; N, blue; Pd, orange. Hydrogens, counterions, and solvent molecules are not shown for clarity.

Single crystals of (*E*-**1**)_2_
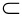
**2** suitable for X-ray diffraction were obtained by slow water evaporation from an aqueous solution of the complex. The data were collected immediately after dipping the crystal in Paratone oil and flash-cooling. We found that (*E*-**1**)_2_
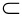
**2** crystallizes in the monoclinic space group *P*2_1_/*c*, with one molecule of **1** and a half-molecule of **2** in the asymmetric unit. In other words, each cage encapsulated two molecules of *E*-**1** (in agreement with the NMR spectra), arranged in an antiparallel fashion (Figure 3). The binding of *E*-**1** within **2** is driven by a combination of π–π stacking, van der Waals forces, and C–H···π interactions (vide infra); in addition, the encapsulation is likely facilitated by the release of "high-energy" water molecules from the cavity of **2** [50–51].

Determining the X-ray structure was challenging; the difficulty lies in the fact that both the cage and the encapsulated guest dimer exist in the form of two different conformations. Figure 3 shows (*E*-**1**)_2_
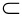
**2** that features the main conformations of both (*E*-**1**)_2_ and **2**, amounting to 59% and 93% of the total population of the guest and the host component, respectively. The remaining 41% of (*E*-**1**)_2_ features the same separation distance between the two guest molecules (*d* = 3.503 Å vs 3.497 Å in the major isomer; a distance typical of π–π-stacked moieties), but a significantly larger offset in the horizontal direction (the distance between *para* carbon atoms of the two guests, *d* = 11.154 Å vs 10.638 Å for the major isomer; see Figure S15, Supporting Information File 1). The difference between the major (93%) and the minor (7%) conformation of the host is small and is mostly related to the positions of the Pd centers (Figure S16, Supporting Information File 1).

We were pleased to find that the structural features of (*E*-**1**)_2_
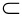
**2** resolved by X-ray crystallography were in full agreement with a detailed analysis of the complex’s NOE spectra (see Figure 2 above). For example, *E*-**1**’s H**_a_** protons at 1.07 ppm correlate with **2**’s axial – but not equatorial – acidic imidazole H**_1_** protons (cyan in Figure 2), which is in agreement with the close proximity of these two protons in the X-ray structure (*d***_a_**_–_**_1_** = 3.68 Å). Conversely, H**_c_** protons only correlate with the equatorial H**_4_** protons (pink in Figure 2; *d***_c_**_–_**_4_** = 3.32 Å). Interestingly, the NOE correlations we found can only be explained by taking into account the coexistence of both configurations of (*E*-**1**)_2_, which suggests that they coexist not only in the crystalline state, but also in solution. For example, H**_c_**’s correlation with the resonance at 7.57 ppm (due to cage’s H**_5_** + H**_7_**) can be explained by the close distance between H**_c_** and H**_5_** in the complex’s minor (41%) isomer, *d***_c_**_–_**_5_** = 2.88 Å (for the major isomer, *d***_c_**_–_**_5_** = 4.10 Å and *d***_c_**_–_**_7_** = 4.13 Å). On the other hand, only the major isomer is expected to show a correlation between H**_a_** and H**_d_** (*d***_a_**_–_**_d_** = 3.38 Å; for the minor isomer, *d***_a_**_–_**_d_** = 4.28 Å). Furthermore, the presence of NOE correlations between H**_d_** and both H**_a_** and H**_c_** not only confirm the presence of two molecules of guest *E*-**1** inside host **2**, but also their antiparallel arrangement.

We also note that the mutual arrangement of the two guest molecules within the X-ray structure is such that the *N*-bound methyl group (i.e., H**_b_** protons) of one guest lies directly above the phenyl ring of the other guest (see Figure S15, Supporting Information File 1). Specifically, the distance between H**_b_** and the plane of the phenyl ring was determined as 2.42 Å, which corresponds to a fairly strong C–H···π interaction. Interestingly, a signature of this interaction can be found in the ^1^H NMR spectrum of (*E*-**1**)_2_
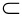
**2**, where the signal due to H**_b_** was significantly broader than those due to H**_a_** and H**_c_** (Figure 1).

**Figure 4:**
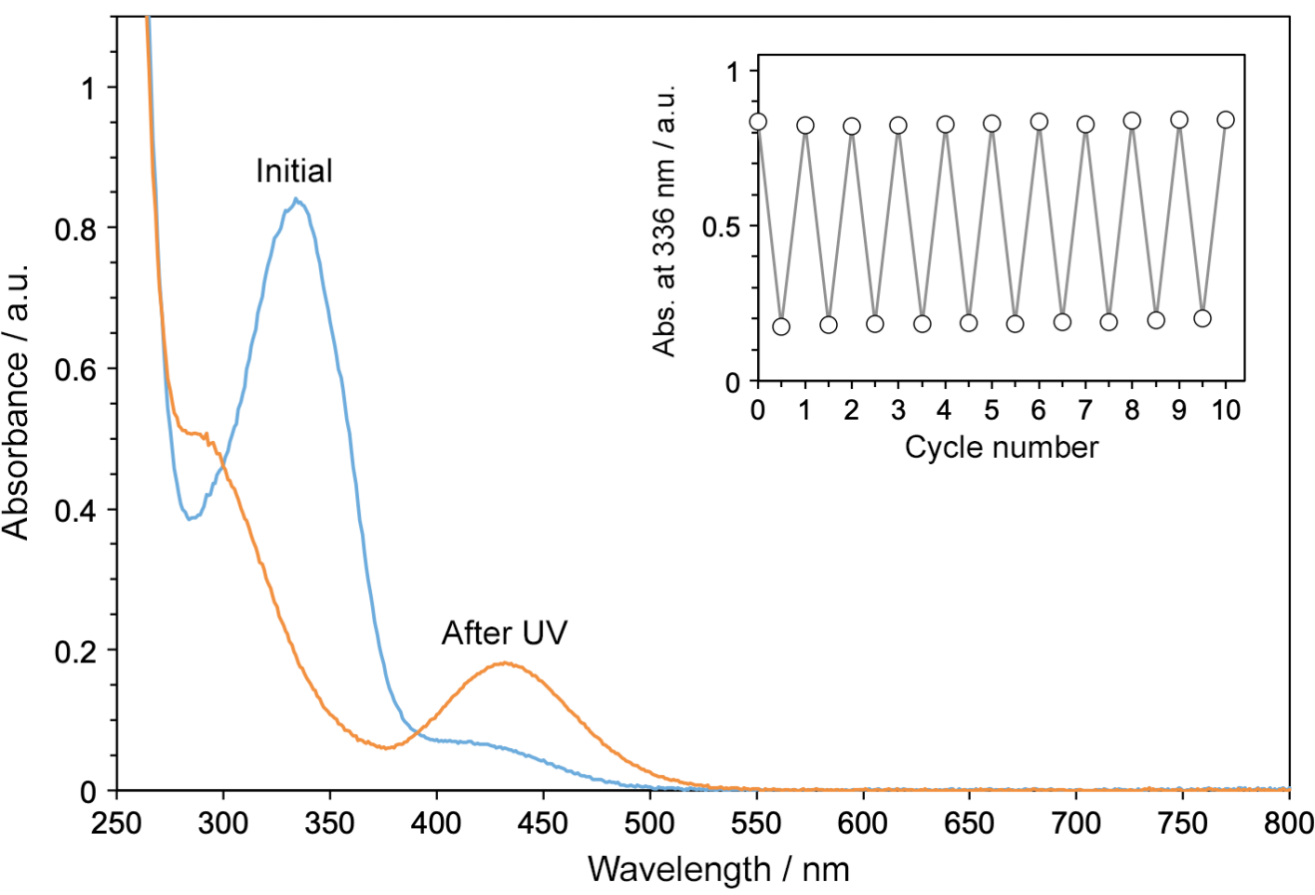
UV–vis absorption spectrum of an aqueous solution of (*E*-**1**)_2_
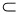
**2** (blue) and following exposure of this solution to UV light for 5 min (orange). Inset: Ten cycles of reversible *E*-**1** ⇌ *Z*-**1** photoisomerization. In each cycle, 5 min of UV was applied, followed by 2 min of green light.

Next, we investigated the photoisomerization of encapsulated *E*-**1**. UV irradiation (365 nm light-emitting diode, LED) of the transparent yellow solution of (*E*-**1**)_2_
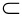
**2** in water did not result in any pronounced visual changes. However, UV–vis absorption spectroscopy showed (Figure 4) a dramatic decrease in the intensity of *E*-**1**’s absorption at 336 nm, along with the concomitant appearance of a broader peak at ~430 nm, which can be attributed to the n–π* transition in *Z*-**1**. Subsequent exposure to a 520 nm LED (a region with negligible absorption of *E*-**1**; see Figure 4) regenerated the initial spectrum, indicating the nearly complete reversibility of the process. The presence of a well-defined isosbestic point (at 391 nm) suggests a clean *E* ⇌ *Z* transformation in both directions. This process appears to be fully reversible for many cycles (Figure 4, inset).

Figure S17 (Supporting Information File 1) shows the ^1^H NMR spectrum of (*E*-**1**)_2_
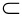
**2** exposed to UV light. Notably, all six resonances of **1**’s protons have shifted, indicating a highly efficient photoisomerization to *Z*-**1**. The corresponding NOE spectra (Figures S18–S20, Supporting Information File 1) feature multiple correlations between the host’s and the guest’s protons. For example, the set of signals at 7.88–7.60 (all of which could be attributed to **2**) correlate strongly with the protons of all three methyl groups of *Z*-**1** (H**_a_**, H**_b_**, and H**_c_**), in addition to exhibiting weaker NOE correlations with H**_d_**, H**_e_**, and H**_f_**. Furthermore, the acidic imidazole protons correlate with most of *Z*-**1**’s protons; the strongest correlations were found between H**_a_** and H**_1_** and between H**_c_** and H**_4_** (Figure S20, Supporting Information File 1; note that the opposite was found for the *E* isomer, cf. Figure 2).

**Figure 5:**
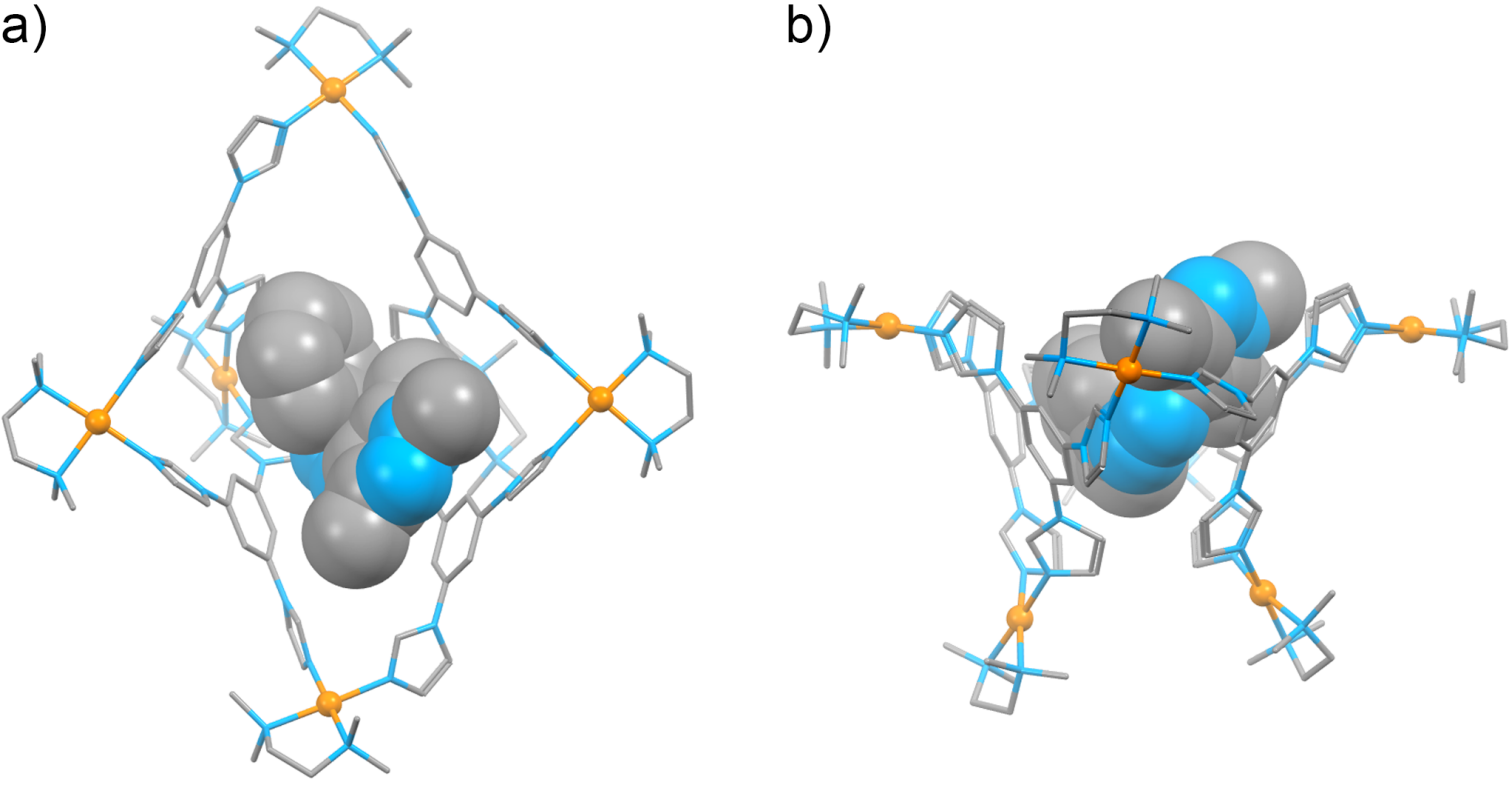
Top view (a) and side view (b) of the energy-optimized structure of (*Z*-**1**)
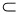
**2**. Color codes: C, gray; N, blue; Pd, orange. Hydrogens and counterions are not shown for clarity.

Despite repeated efforts, we did not succeed in preparing crystals of the (*Z*-**1**)
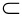
**2** inclusion complex suitable for X-ray diffraction. To obtain hints about the packing of *Z*-**1** within **2**, we therefore performed density functional theory (DFT) calculations. Compared with the complex before photoisomerization, the cage in (*Z*-**1**)
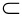
**2** is severely distorted (Figure 5; see also Supporting Information File 2), assuming a bowl-like shape (in contrast, the conformation of the bound guest is very similar to that of free *Z*-**1**). The optimized model of (*Z*-**1**)
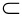
**2** features strong interactions between the host and the guest. In particular, *Z*-**1**’s methyl groups H**_a_** and H**_c_** reside in close proximity to **2**’s acidic imidazole protons (H**_1_** and H**_4_**, respectively), in agreement with the NMR findings (see Section 4 in the Supporting Information File 1). The apparent desymmetrization of **2** is not manifested by multiplication of signals due to H**_1_** and H**_4_**, which can be attributed to the dynamic fluctuations of the cage in solution.

We have previously noted that 1:1 complexes of **2** with the *Z* isomers of various azobenzenes are notoriously difficult to crystallize [18]. We believe that the insights by DFT calculations reported here for (*Z*-**1**)
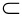
**2** can be extended to the complexes of **2** with other *Z*-azo guests as well.

**Figure 6:**
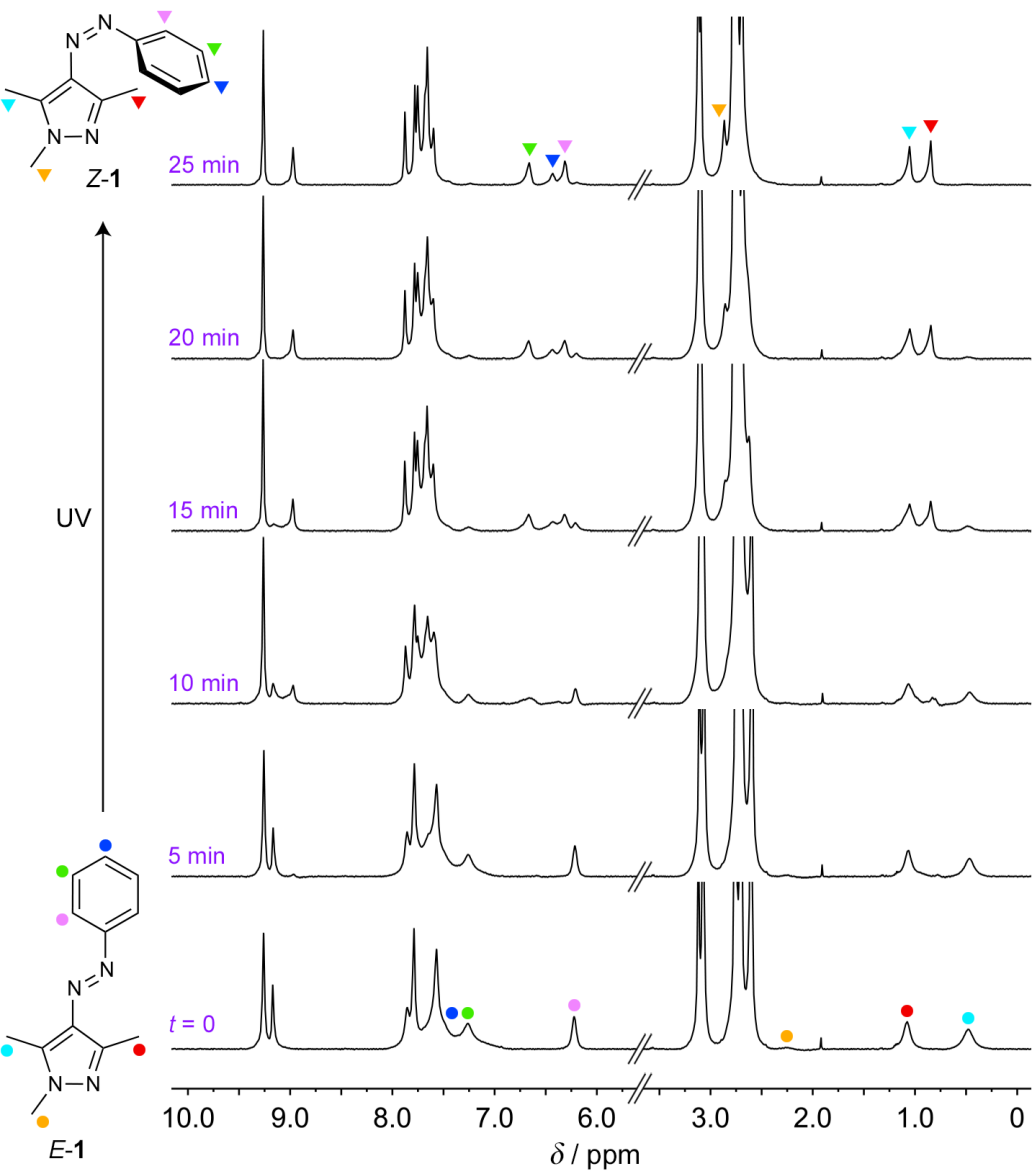
A series of ^1^H NMR spectra of (*E*-**1**)_2_
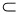
**2** (500 MHz, D_2_O, 298 K) before (bottom) and after exposure to UV light (λ = 365 nm) inside the NMR spectrometer (using an optical fiber) for different periods of time (indicated in purple font) for up to 25 min.

To gain further insight into the photoisomerization reaction, we investigated it by NMR spectroscopy under in-situ irradiation (with an optical fiber inserted into the NMR spectrometer). Figure 6 shows a series of spectra recorded after increasing UV irradiation times. We found that whereas the position of **2**’s H**_4_** signal did not change (9.26 ppm), the signal due to H**_1_** shifted upfield by 0.20 ppm. This behavior is analogous to our previous findings, which showed that shifts of H**_1_** protons are indicative of switching of the bound guest and/or (de)encapsulation [18,49]. Integrating the signals due to *E*-**1**’s H**_c_** protons (cyan in Figure 6) allowed us to estimate the yield of photoisomerization as >98%. This result, combined with the UV–vis absorption data in Figure 4, inset, indicates that the switching between *E*- and *Z*-**1** is near-quantitative in both directions.

To complete the cycle, we first allowed the system to relax thermally while recording NMR spectra (Figure S21, Supporting Information File 1). However, only 25% of *Z*-**1** back-isomerized to the *E* isomer within 3 h in the dark. Subsequent irradiation with green light (20 min) re-generated the initial solution of (*E*-**1**)_2_
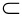
**2**. Interestingly, the final spectrum, although virtually free of *Z*-**1**, exhibited a residual (≈7%) peak of **2** with a conformation that binds *Z*-**1** (see Figure S22, Supporting Information File 1, red arrow at 8.97 ppm). This result suggests that the *Z*-**1** → *E*-**1** back-isomerization is faster than the conformational change of **2**, and that the cage gradually adapts its shape to that of the bound guest.

Further analysis of the NOE spectra of UV-irradiated solutions revealed correlations between *Z*-**1**’s i) H**_a_** and H**_d_**, ii) H**_a_** and H**_e_**, iii) H**_c_** and H**_d_**, and iv) H**_c_** and H**_e_** (all expected from the *Z* isomer alone), but no correlations between i) H**_b_** with any of the three aromatic protons and ii) H**_f_** with any of the three methyl groups. Furthermore, we note that the resonance due to H**_b_** – very broad in complex (*E*-**1**)_2_
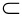
**2** as a consequence of dimer formation – becomes as sharp as those of the other protons after UV irradiation (compare spectra **i** and **ii** in Figure S23, Supporting Information File 1). Taken together, these observations indicate the absence of *Z*-**1**–*Z*-**1** interactions, thereby confirming that only one molecule of *Z*-**1** occupies the cavity of the cage. This conclusion is not surprising given the more bulky nature of the *Z* isomer – in fact, we have previously reported that photoirradiation of the 1:2 complex of **2** and tetra-*o*-fluoroazobenzene yields a 1:1 mixture of *Z*
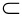
**2** and free *Z*-azobenzene, which is insoluble in water and precipitates from the solution [49]. The *Z* isomer of **1**, however, has much higher water solubility and, following expulsion from the cage, can remain stable in solution. To verify this notion, we prepared pure *Z*-**1** (by evaporating the solvent from a solution of *E*-**1** in acetonitrile under continuous UV irradiation) and i) confirmed its high solubility in water (see Figure S24 in Supporting Information File 1 for an NMR spectrum of *Z*-**1** in D_2_O) and ii) demonstrated light-controlled precipitation and re-solubilization of **1** (Figure S25, Supporting Information File 1).

Overall, the reaction taking place in the system can be written down as (*E*-**1**)_2_
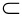
**2** → (*Z*-**1**)
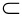
**2** + *Z*-**1**. Interestingly, however, despite the existence of *Z*-**1** as two distinct species (encapsulated and non-encapsulated), the NMR spectrum of the UV-adapted solution showed the presence of only one set of guest peaks. This can be attributed to the rapid (on the NMR time scale) exchange between the free and encapsulated *Z*-**1**, resulting in a single peak for each proton, located in between the two expected peaks. To confirm this hypothesis, we recorded ^1^H NMR spectra of i) *Z*-**1** in D_2_O (spectrum **i** in Figure S27) and ii) (*Z*-**1**)
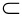
**2** without free *Z*-**1** (**ii** in Figure S27, Supporting Information File 1) (the latter was prepared by treating a UV-irradiated solution of (*E*-**1**)_2_
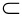
**2** (i.e., a mixture of (*Z*-**1**)
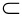
**2** and *Z*-**1**) with an extra ~2.5 equivalents of **2**). Indeed, the resonances due to all **1**’s protons in (*E*-**1**)2
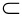
**2** exposed to UV light (Figure S26, Supporting Information File 1, spectrum **iii**) appeared at chemical shifts that constituted the averages of those found in spectra **i** and **ii**. As a control experiment, we added the same excess of **2** to a solution of (*E*-**1**)_2_
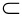
**2** prior to UV irradiation and, as expected, did not observe any shifts of **1**’s signals.

**Figure 7:**
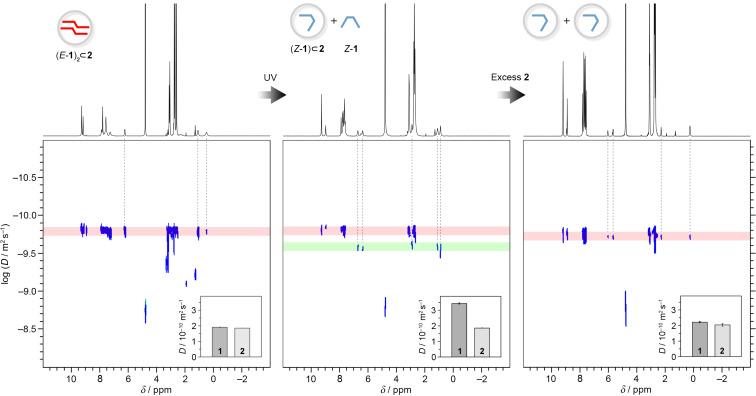
^1^H NMR and ^1^H DOSY spectra of (*E*-**1**)_2_
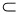
**2** (500 MHz, D_2_O, 298 K) before (left) and after (center) UV irradiation and after the addition of ~2.5 equiv of **2** (right). The vertical dashed lines denote the resonances of **1**’s protons.

To further confirm the photoswitching-induced guest expulsion, we performed detailed diffusion-ordered spectroscopy (DOSY) measurements. A representative DOSY spectrum of (*E*-**1**)_2_
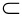
**2** is shown in Figure 7, left. The spectrum indicates the presence of a single species (the diffusion coefficient for **1**, *D***_1_** = 1.91 (±0.01) · 10^−10^ m^2^·s^−1^ (based on averaging H**_a_** and H**_d_**); the diffusion coefficient for **2**, *D***_2_** = 1.85 (±0.01) · 10^−10^ m^2^·s^−1^ (based on H**_4_**); each diffusion measurement was repeated three times). In contrast, DOSY spectra recorded after UV irradiation exhibited two distinct sets of diffusion coefficients, associated with resonances of **1** and **2** (red and green shades in Figure 7, center). The average diffusion coefficients were determined as *D***_1_** = 3.44 (±0.04) · 10^−10^ m^2^·s^−1^ and *D***_2_** = 1.86 (±0.01) · 10^−10^ m^2^·s^−1^. The greatly increased *D***_1_** value confirms that i) **1** was partially expelled from the cages, which can only accommodate one molecule of *Z*-**1** each, and ii) the free and bound *Z*-**1** are in rapid exchange with each other. Finally, the addition of enough cages to re-encapsulate all the expelled *Z*-**1** results in a spectrum once again featuring a single set of diffusion coefficients (Figure 7, right; *D***_1_** = 2.22 (±0.04) · 10^−10^ m^2^·s^−1^ and *D***_2_** = 2.04 (±0.08) · 10^−10^ m^2^·s^−1^).

**Figure 8:**
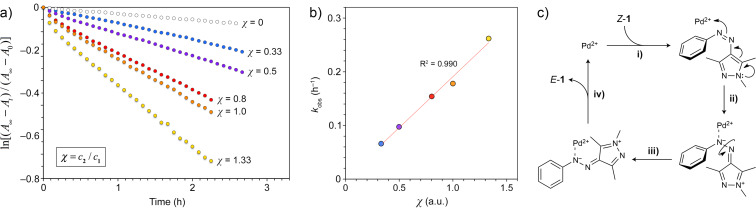
Palladium-accelerated back-isomerization of *Z*-**1**. a) Kinetics of the thermal back-isomerization of *Z*-**1** (*c* = 0.187 mM) in the presence of increasing concentrations of **2** (*A*_∞_ denotes the absorbance at λ_max_ before irradiation; *A*_0_ – immediately after exposure to UV light; *A*_t_ – after thermal relaxation for time *t*). b) Cage concentration dependence of the pseudo-first-order rate constant *k*_obs_. c) The proposed mechanism underlying the Pd^2+^-accelerated back-isomerization of *Z*-**1**.

Finally, we were interested in how the presence of cage **2** affects the kinetics of the thermal back-isomerization of **1** (i.e., *Z*-**1** → *E*-**1** in the dark). This reaction was followed by monitoring the recovery of absorbance at 336 nm, which corresponds to the maximal absorbance (λ_max_) of the *E* isomer. The orange circles in Figure 8a show the recovery profile of *E*-**1** in the presence of 0.5 equiv of **2**, following the reaction equation, (*Z*-**1**)
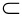
**2** + *Z*-**1** → (*E*-**1**)_2_
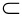
**2**. The linear profile of the recovery suggests that the reaction obeys first-order kinetics, with a rate constant of 0.0975 h^−1^, corresponding to a thermal half-life of *Z*-**1**, *τ*_1/2_ ≈ 7.1 hours. This value of *τ*_1/2_ is surprisingly small vis-à-vis the previously reported [35] *τ*_1/2_ ≈ 10 days in acetonitrile. To verify that this dramatic destabilization of *Z*-**1** does not reflect the effect of the solvent, we independently studied back-isomerization in an aqueous solution (Figure 8a, empty circles) and found that the presence of water does promote the *Z*→*E* reaction (*τ*_1/2_ ≈ 27.9 hours), although to a much lesser extent than does aqueous **2**.

These results suggest that cage **2** can catalyze the thermal back-isomerization of *Z*-**1** to *E*-**1**, whose kinetics can be written down as:

[1] $$-\frac{\partial c_{Z\text{-}1}}{\partial t}=kc_{Z\text{-}1}c_{2}=k_{\text{obs}}c_{Z\text{-}1},$$

where the pseudo-first-order rate constant *k*_obs_ is the product of *k* and the concentration of the cage, *c***_2_**, which remains constant over time. To validate this model, we studied the kinetics of *Z*→*E* back-isomerization in the presence of various concentrations of the cage (blue, purple, red, and yellow circles in Figure 8a) and found that *k*_obs_ indeed scales roughly linearly with *c***_2_** (Figure 8b).

We hypothesized that the acceleration effect may be due to Pd^2+^ ions residing at the corners of **2**. Although all Pd^2+^s within the cage are coordinately saturated, **2** may exist in equilibrium with a small amount of a species, in which one of three imidazole groups of a triimidazole panel is temporarily dissociated from Pd^2+^, enabling its interaction with *Z*-**1** (Figure 8c, step **i**). Coordination of Pd^2+^ to the azo moiety, combined with the strongly electron-donating pyrazole moiety, affords (Figure 8c, step **ii**) a system akin to push–pull azobenzenes, whose *Z* isomers can back-isomerize rapidly due to a reduced order of the N=N bond [52–54] (Figure 8c, step **iii**). The resulting *E*-**1** can be liberated and the Pd^2+^ center is available to carry out the cycle on another molecule of *Z*-**1** (Figure 8c, step **iv**). This reasoning is supported by the observation that Pd^2+^ intentionally added to a solution of *Z*-**1** (in the form of [Pd(NO_3_)_2_(tmeda)]; 0.944 mM) also induces a fast back-isomerization reaction, with *k*_obs_ ≈ 0.255 h^−1^. The fast acceleration in the presence of **2** – despite low concentrations of the active Pd species – is likely due to the fast encapsulation of *Z*-**1** in **2**, which greatly increases the local concentration of Pd^2+^. Indeed, when *Z*-**1** was mixed with a premade complex of **2** with a tightly bound guest, tetra-*o*-fluoroazobenzene [49], no acceleration effect was observed.

## Conclusion

In summary, we investigated the encapsulation and switching of a prototypical arylazopyrazole in the hydrophobic cavity of a water-soluble metal–organic cage. A host–guest inclusion complex incorporating two molecules of arylazopyrazole was obtained in a quantitative yield. The complex was characterized by a series of NMR techniques, which showed that the resonances of the guest protons were upfield-shifted by up to 2.2 ppm. An antiparallel configuration of the guest molecules inside the cage was demonstrated independently by NMR spectroscopy and single-crystal X-ray diffraction. Exposure to UV light resulted in an *E*→*Z* isomerization of the guest, which was accompanied by the expulsion of one of two guest molecules from the cage. Different from the previously reported azobenzene complex, the photoisomerization was not accompanied by precipitation of the released *Z*-arylazopyrazole, which has good water solubility. The expulsion of the *Z* isomer was confirmed by diffusion-ordered NMR spectroscopy, which also showed that the released and the encapsulated *Z*-arylazopyrazole were in rapid exchange with each other. The back-isomerization reaction was achieved by irradiation with green light and it was accompanied by regeneration of the initial 2:1 complex; the *E* ⇌ *Z* transformation proceeded in a near-quantitative fashion in both directions. The back-isomerization also proceeded thermally in a reaction that was accelerated by the cage. The acceleration could be attributed to the coordination of Pd^2+^ to the N=N moiety, as reported previously for azobenzene back-isomerization in the presence of Cu^2+^ [55] and for *Z*-arylazopyrazoles in the presence of an acid [56]. Overall, the results reported here strengthen our understanding of the behavior of molecular switches in confined spaces.

## Experimental

**Synthesis of cage 2:** Cage **2** was prepared based on a modified literature known procedure [18]. Briefly, a solution of [Pd(NO_3_)_2_(tmeda)] (200 mg, 0.577 mmol) in 25 mL of water was slowly added to 1,3,5-tri(1*H*-imidazol-1-yl)benzene (106 mg, 0.384 mmol) and the resulting mixture was stirred for 24 h at room temperature. Then, all the insoluble materials were removed by centrifugation and the supernatant was concentrated in vacuo. Crystalline **2** was obtained by acetone vapor diffusion to an aqueous solution of **1** (290 mg; yield = 95%). Cage **2** has an excellent solubility in water and is stable in the pH range of 4–9. It also exhibits high thermal stability, remaining stable for prolonged periods at 80 °C.

**Synthesis of inclusion complex (*E*-1)_2_**
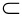
**2:** *E*-**1** was encapsulated by stirring excess solid *E*-**1** with a solution of **2** in water (H_2_O/D_2_O). Encapsulation of *E*-**1** by **2** was followed by UV–vis absorption spectroscopy; stirring was discontinued when no further coloration of the solution was observed. Excess *E*-**1** was removed by filtration. ^1^H NMR (500 MHz, D_2_O) δ 9.26 (s, 8H), 9.17 (s, 4H), 7.85 (s, 4H), 7.78 (br, 8H), 7.57 (br, 18H), 7.25 (br, 12H), 6.22 (br, 4H), 3.12 (s, 8H), 3.07 (s, 16H), 2.76 (s, 24H), 2.70 (s, 24H), 2.60 (s, 24H), 2.29 (s, 6H), 1.07 (s, 6H), 0.47 (s, 6H); ^13^C{^1^H} NMR (125 MHz, D_2_O) δ 152.0, 138.8, 138.3, 138.1, 137.1, 133.2, 131.7, 130.5, 130.4, 129.8, 121.6, 120.9, 120.5, 112.9, 111.8, 63.3, 63.2, 51.0, 50.9, 50.7, 34.6, 12.5, 7.3 (the signal assignment is based on COSY, NOESY, HSQC, and HMBC spectra of the complex, as well as COSY, NOESY, and HSQC spectra of free *E*-**1** recorded in CDCl_3_).

**Thermal back-isomerization studies:** The *Z* isomer of **1** was first prepared by UV irradiation of a solution of *E*-**1** in acetonitrile, followed by solvent evaporation under UV light. *Z*-**1** was dissolved in pure water and a desired amount of an aqueous solution of cage **2** was added (final concentration of *Z*-**1** = 0.187 mM; *c***_2_** = 0.0623 mM (0.33 equiv); 0.0935 mM (0.5 equiv); 0.150 mM (0.8 equiv), 0.187 mM (1.0 equiv); 0.249 mM (1.33 equiv)). The rate of back-isomerization was followed by monitoring the increase in absorbance at λ_max_ (336 nm). In control experiments, we followed the back-isomerization of *Z*-**1** (0.187 mM) in the absence of **2** and in the presence of [Pd(NO_3_)_2_(tmeda)] (0.944 mM) and a 1:2 complex of **2** and tetra-*o*-fluoroazobenzene [49].

## Supporting Information

The Supporting Information features further experimental details, including synthesis and characterization of arylazopyrazole **1**, characterization of inclusion complexes (*E*-**1**)_2_
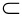
**2** and (*Z*-**1**)
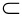
**2**, photoisomerization and thermal relaxation studies, and details on X-ray data collection and refinement. In addition, X-ray data for inclusion complex (*E*-**1**)_2_
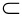
**2** and the energy-optimized structure for inclusion complex (*Z*-**1**)
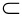
**2** are given.

File 1 Further experimental details.

File 2X-ray data for inclusion complex (*E*-**1**)_2_
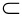
**2**.

File 3Energy-optimized structure for inclusion complex (*Z*-**1**)
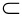
**2**.
