# Supplementary material for: Reversible switching of arylazopyrazole within a metal–organic cage
Source: Beilstein J Org Chem. 2019 Oct 10;15:2398–407. doi: 10.3762/bjoc.15.232 (PMC6808206; doi:10.3762/bjoc.15.232)
Supplement: File 1 — Further experimental details. [file Beilstein_J_Org_Chem-15-2398-s001.pdf]

BEILSTEIN JOURNAL OF ORGANIC CHEMISTRY

Supporting Information

for

Reversible switching of arylazopyrazole within a metal–organic cage

Anton I. Hanopolskyi, Soumen De, Michał J. Białek, Yael Diskin-Posner, Liat Avram, Moran Feller and Rafal Klajn

Beilstein J. Org. Chem. **2019,** *15,* 2398–2407. doi:10.3762/bjoc.15.232

Further experimental details

License and Terms: This is a supporting information file under the terms of the Creative Commons Attribution License (http://creativecommons.org/licenses/by/4.0). Please note that the reuse, redistribution and reproduction in particular requires that the authors and source are credited.
The license is subject to the *Beilstein Journal of Organic Chemistry* terms and conditions: (https://www.beilstein-journals.org/bjoc)

Table of contents

1. General information S1
2. Synthesis of arylazopyrazole **1** S1
3. Synthesis of cage **2** S3
4. Characterization of inclusion complex (E-**1**)$_2$⊂**2** S11
5. X-ray data collection and refinement S17
6. DFT calculations S18
7. Supplementary references S19

1. General information

All commercial chemicals were used as received. Guest **1** and cage **2** were prepared following modified literature procedures. NMR spectra were recorded on a Bruker Avance III 300 MHz or on Bruker Avance III HD 500 MHz spectrometer. Chemical shifts (δ) are given in ppm relative to residual protio solvent resonances (4.79 ppm for D_2O and 7.26 ppm for $CDCl_3$). A constant temperature of 298 K was maintained during the measurements. UV/Vis absorption spectra were recorded with a Shimadzu UV-2700 or a UV-3600 spectrophotometer. For photoirradiation experiments, we used a Prizmatix Mic-LED 365 nm light-emitting diode (LED) as a UV light source and a Prizmatix 520 nm Ultra High Power (UHP) Mic-LED LED (collimated LED power of 900 mW) as a green light source. For studying the photoisomerization reactions in-situ using NMR spectroscopy, the LEDs were equipped with a high numerical aperture polymer optical fiber (POF) (diameter 1 mm, length 5 m), which was inserted into the NMR spectrometer. For details on X-ray data collection and refinement, see Section 5. For details on DFT calculations, see Section 6.

2. Synthesis of arylazopyrazole **1**

Arylazopyrazole **1** was synthesized by modifying a previously published procedure [1].

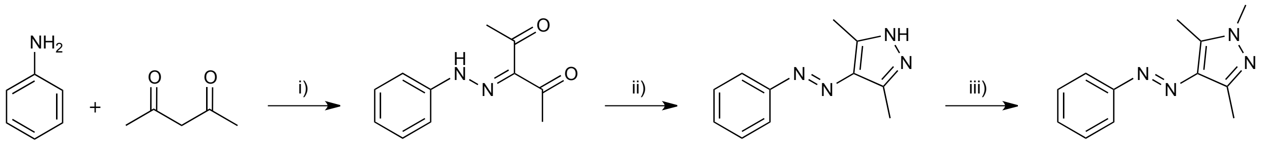

Scheme S1. Synthetic route for arylazopyrazole **1**. Reagents and conditions: i) $NaNO_2$, HCl, EtOH/H_2O, 0-5 ºC, 78%; ii) $N_2H_4{\cdot}H_2O$, EtOH, reflux, 91%; iii) CH_3I, K_2CO_3, DMF, 90%.

3-(2-phenylhydrazono)pentane-2,4-dione (3): A solution of aniline (0.5 g; 5.4 mmol) in 1 M aqueous HCl (10 mL) was cooled to 0-5 ºC and $NaNO_2$ (0.38 g; 5.6 mmol) was added. After 1 h, an ice-cold solution of pentane-2,4-dione (0.67 g; 0.67 mmol) in 4 mL of 5:3 *v*/*v* water–ethanol was slowly added. After 2 h, aqueous NH_4OAc was added until pH = 5-6, resulting in precipitation of the product, which was collected by filtration and dried *in vacuo* overnight (0.85 g, yield = 78%).

^{1}H NMR (300 MHz, $CDCl_3$): δ = 7.43–7.21 (m, 5H), 2.61 (s, 3H), 2.50 (s, 3H).

3,5-dimethyl-4-(phenyldiazenyl)-1*H*-pyrazole (4): To a solution of **3** (0.5 g; 2.4 mmol) in ethanol was added hydrazine monohydrate (110 mg; 2.2 mmol) and the mixture was refluxed for 6 h. Then, the volatiles were removed *in vacuo*, resulting in 0.46 g of **4** (yield = 91%).

^{1}H NMR (300 MHz, DMSO-d_6): δ = 7.72 (d, 2H), 7.54 (t, 2H), 7.44 (t, 1H), 2.52 (s, 6H).

1,3,5-trimethyl-4-(phenyldiazenyl)-1H-pyrazole (**1**): To a solution of **4** (0.46 g; 2.3 mmol) in 2 mL of DMF were added sequentially K_2CO_3 (0.95 g; 6.9 mmol) and CH_3I (0.327 g; 2.3 mmol) and the resulting mixture was stirred overnight at room temperature. Then, 20 mL of H_2O was added and the product was extracted with ethyl acetate (20 mL × 3). The organic phase was washed with brine, dried with $MgSO_4$, and concentrated *in vacuo*, resulting in 0.44 g of **1** as a yellow powder (yield = 90%).

^{1}H NMR (500 MHz, $CDCl_3$): δ = 7.78 (d, 2H), 7.46 (t, 2H), 7.36 (t, 1H), 3.78 (s, 3H), 2.58 (s, 3H), 2.50 (s, 3H).

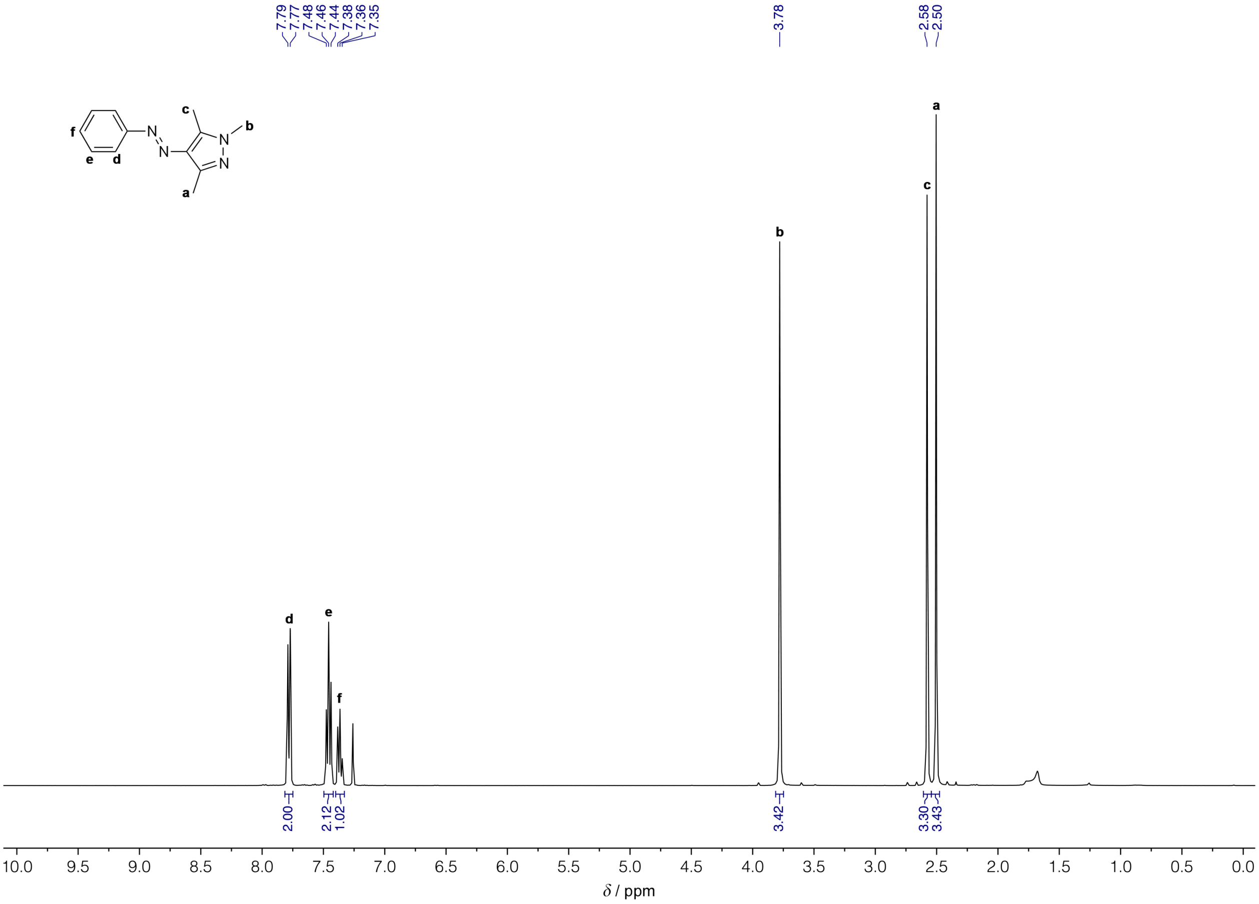

Figure S1: ^{1}H NMR spectrum of *E*-**1** (500 MHz, $CDCl_3$, 298 K).

3. Characterization of inclusion complex $(E\text{-}\mathbf{1})_2{\subset}\mathbf{2}$

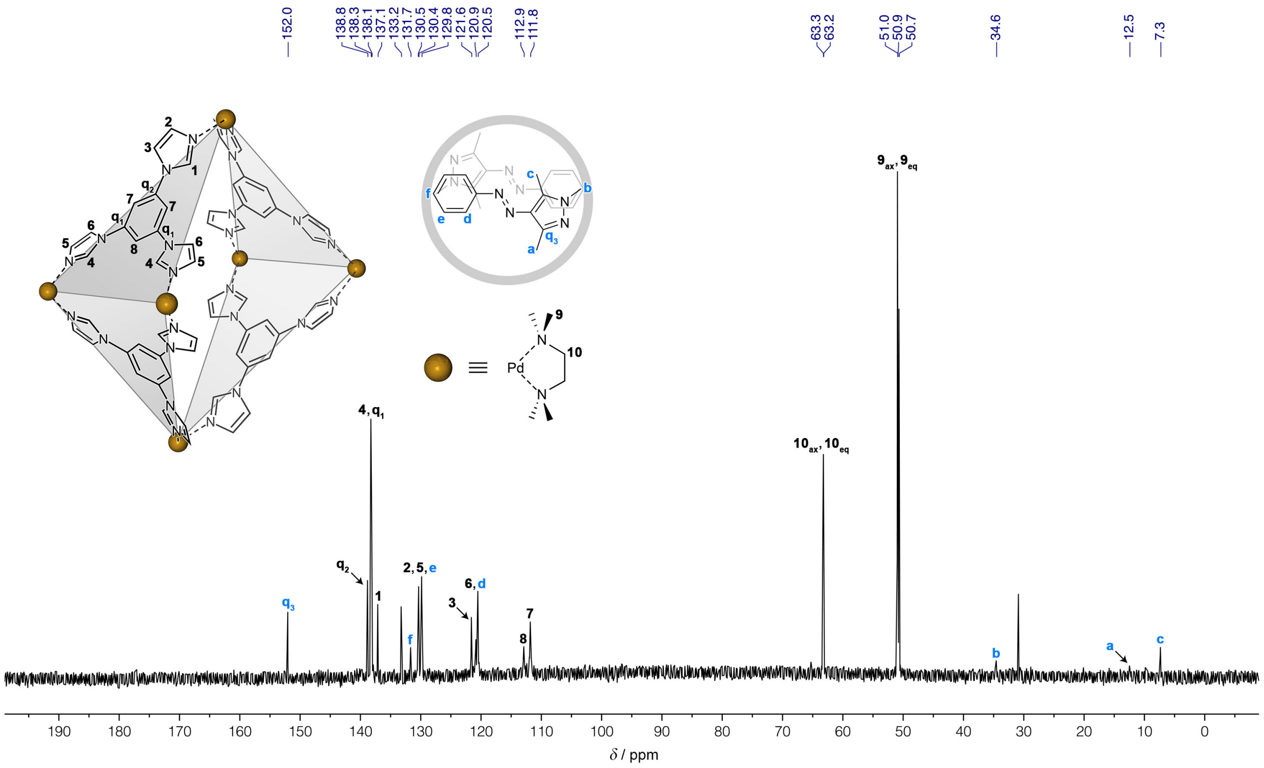

Figure S2: $^{13}C\{^1H\}$ NMR spectrum of $(E\text{-}\mathbf{1})_2{\subset}\mathbf{2}$ (125 MHz, D_2O, 298 K). The resonance at 30.9 ppm is due to residual acetone. The resonance at 133.2 ppm is due to one of the quaternary carbons of encapsulated *E*-**1**.

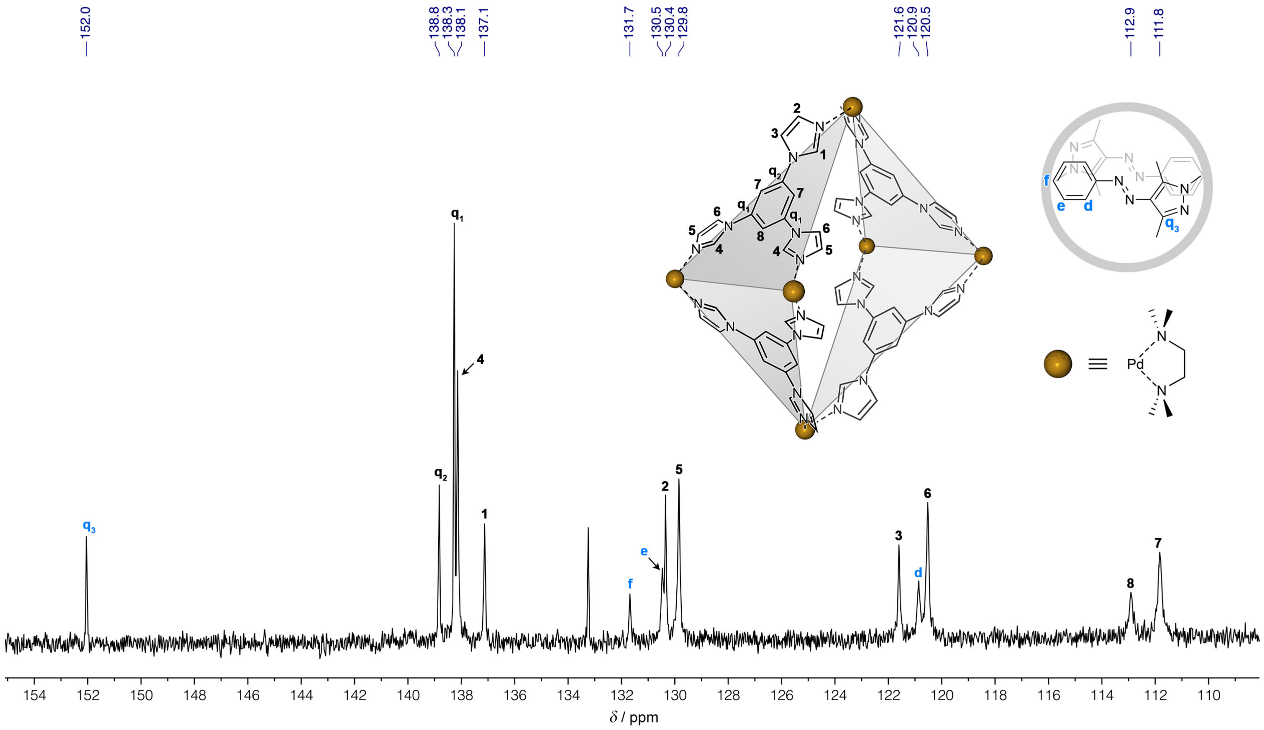

Figure S3: Partial $^{13}C\{^1H\}$ NMR spectrum of $(E\text{-}\mathbf{1})_2{\subset}\mathbf{2}$ (125 MHz, D_2O, 298 K).

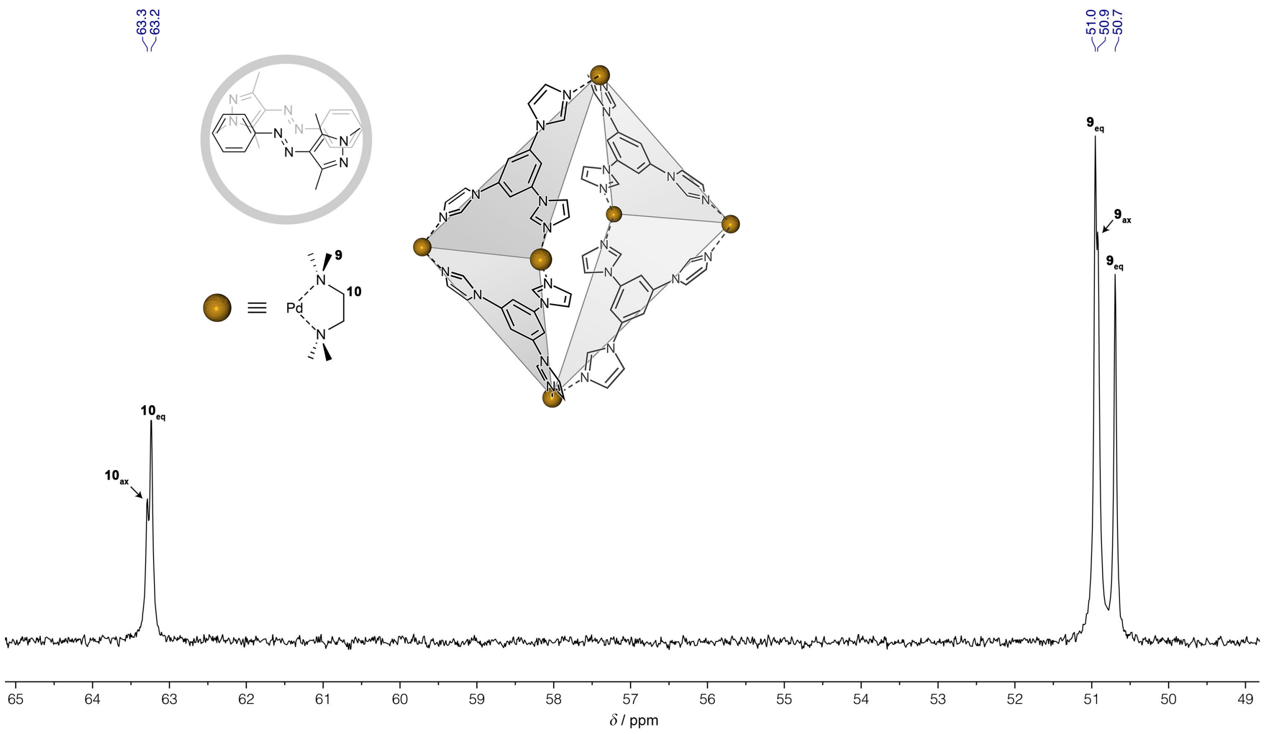

Figure S4: Partial $^{13}C\{^1H\}$ NMR spectrum of $(E\text{-}\mathbf{1})_2{\subset}\mathbf{2}$ (125 MHz, D_2O, 298 K).

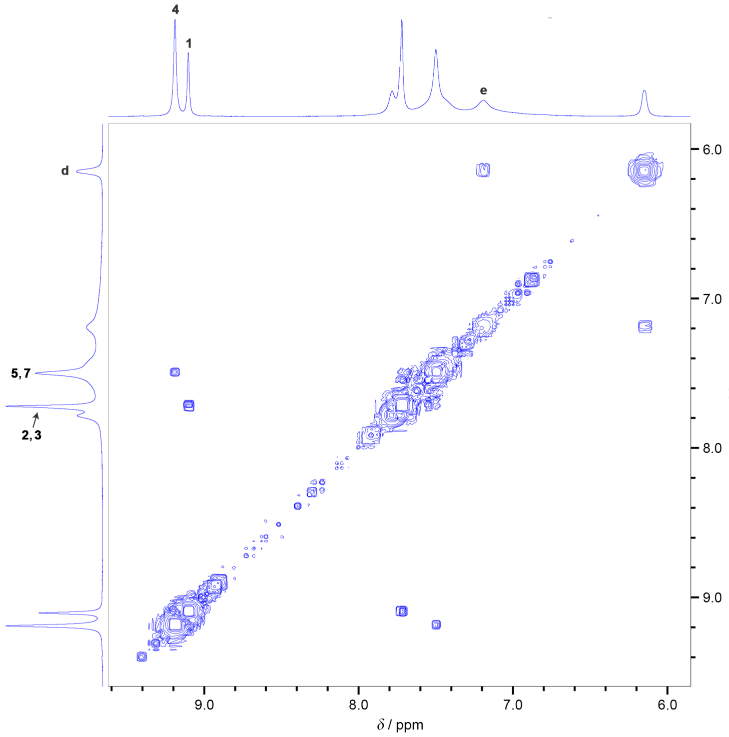

Figure S5: Partial ^1H-^1H COSY NMR spectrum of $(E\text{-}\mathbf{1})_2{\subset}\mathbf{2}$ (500 MHz, D_2O, 298 K).

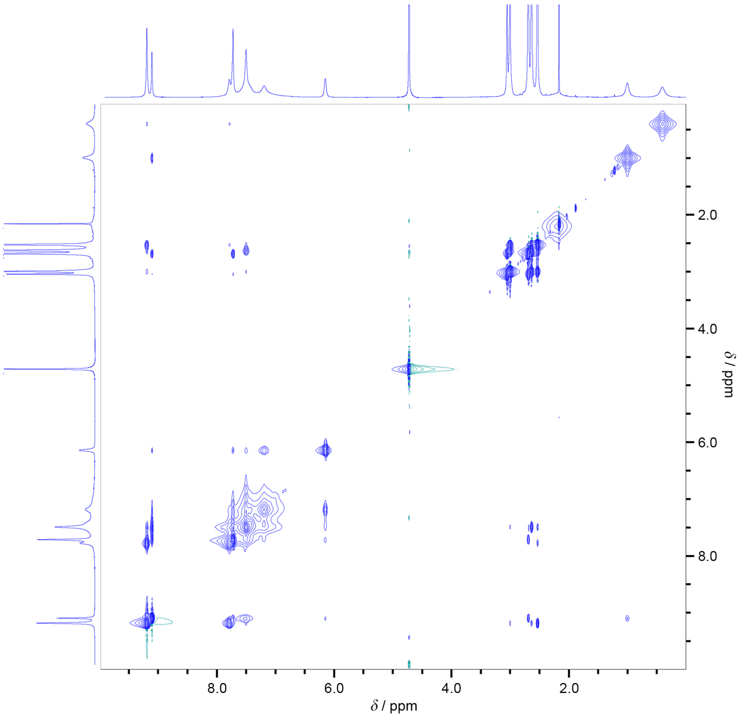

Figure S6: ^{1}H-^{1}H NOESY NMR spectrum of (*E*-**1**)$_2$⊂**2** (500 MHz, D_2O, 298 K). The resonance at 2.22 ppm is due to residual acetone (note that it overlaps with the broad peak at 2.29 ppm due to *E*-**1**'s proton **b**).

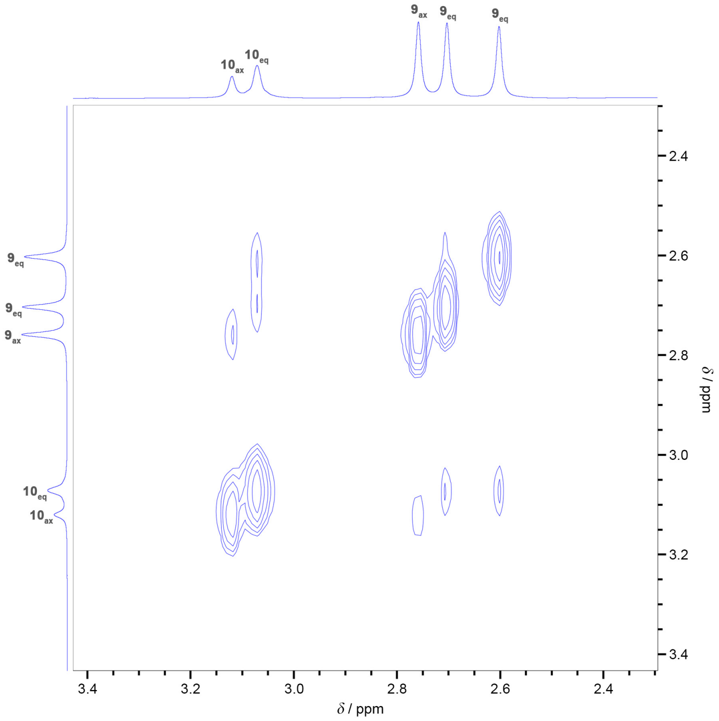

Figure S7: Partial ^{1}H-^{1}H NOESY NMR spectrum of (*E*-**1**)$_2$⊂**2** (500 MHz, D_2O, 298 K) (the corresponding full-range spectrum is shown in Figure S6).

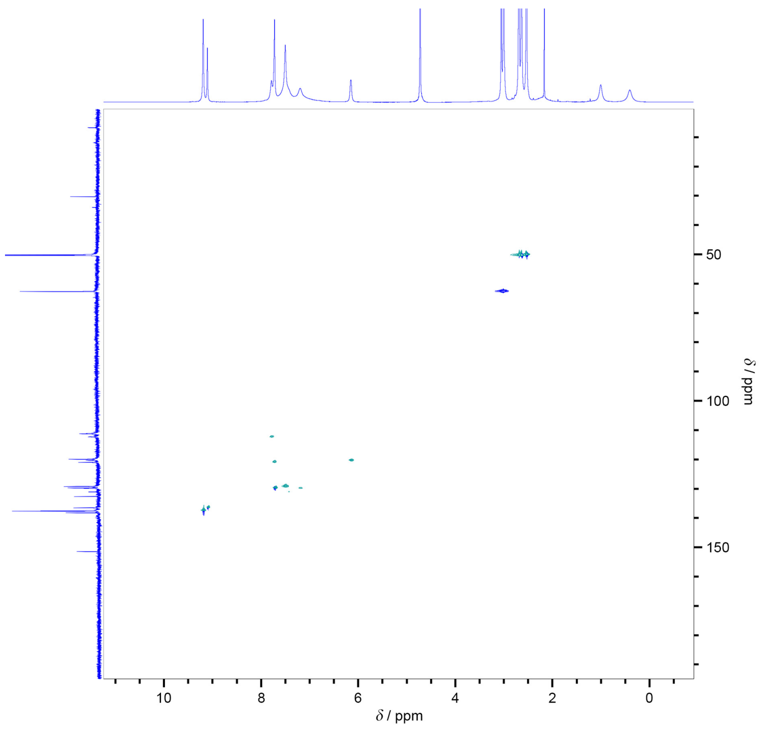

Figure S8: ^{1}H-^{13}C HSQC NMR spectrum of (E-**1**)$_2$⊂**2** (500 MHz, D_2O, 298 K).

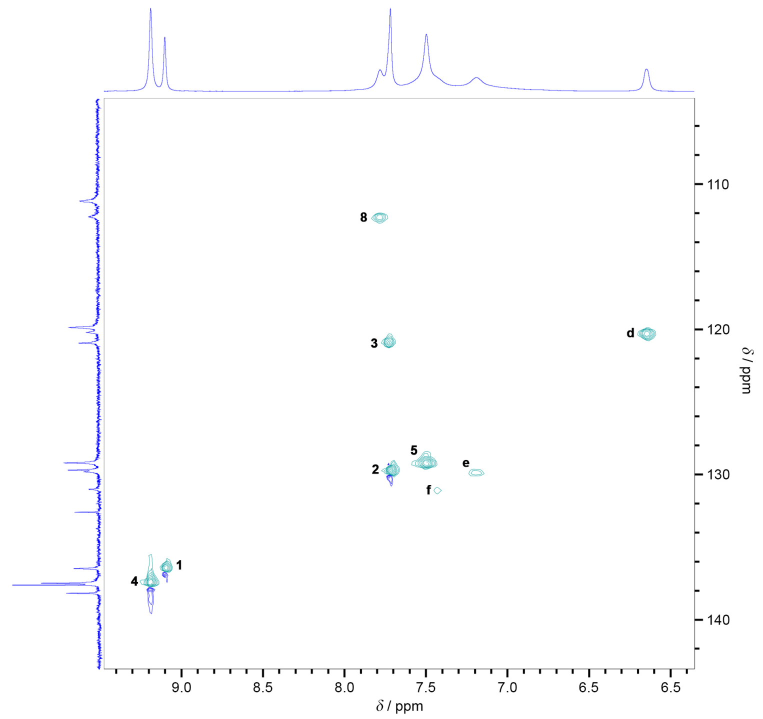

Figure S9: Partial ^{1}H-^{13}C HSQC NMR spectrum of (E-**1**)$_2$⊂**2** (500 MHz, D_2O, 298 K) (the corresponding full-range spectrum is shown in Figure S8). Correlation between H_7 (7.57 ppm) and C_7 at 111.8 ppm was found in other HSQC spectra recorded on the same sample.

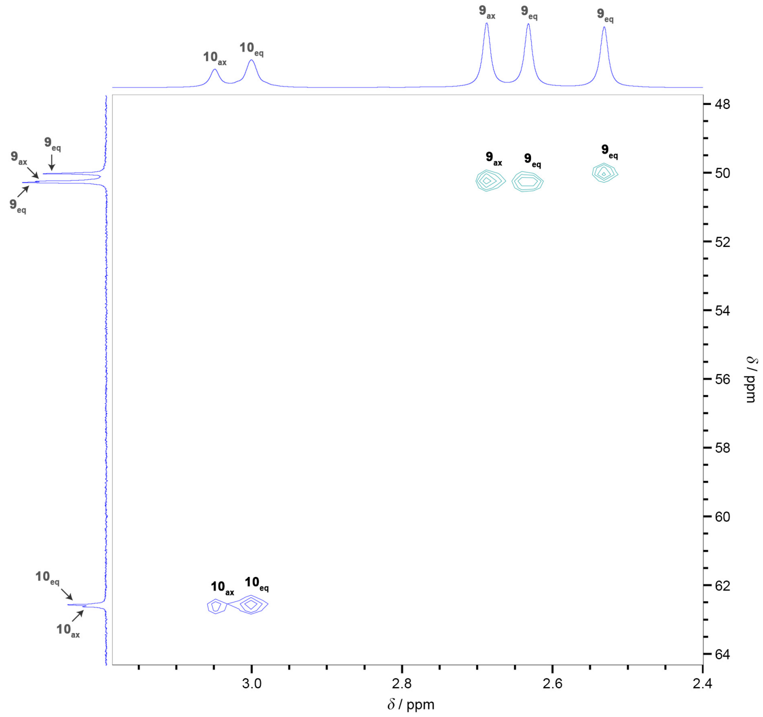

Figure S10: Partial ^{1}H-^{13}C HSQC NMR spectrum of (E-**1**)$_2$⊂**2** (500 MHz, D_2O, 298 K) (the corresponding full-range spectrum is shown in Figure S8).

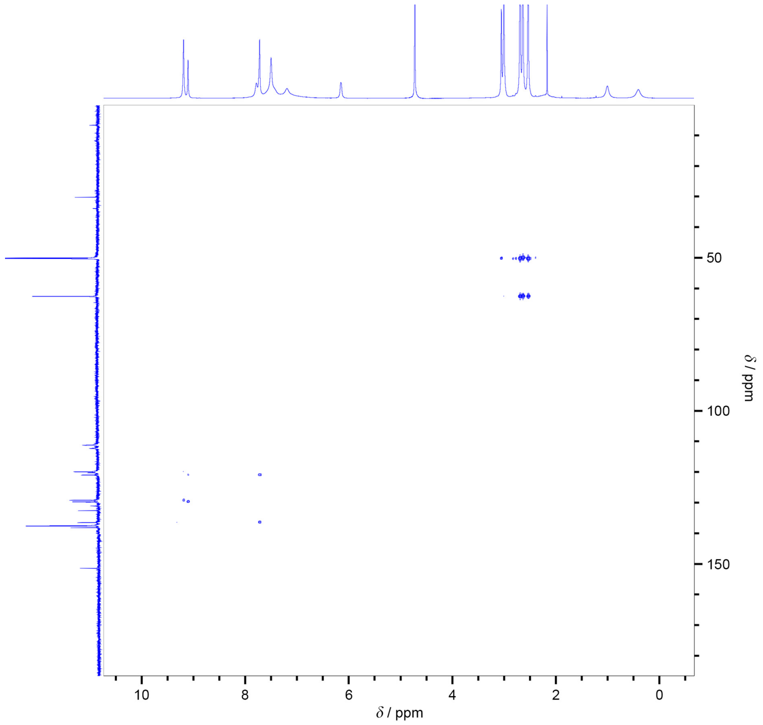

Figure S11: ^{1}H-^{13}C HMBC NMR spectrum of (E-**1**)$_2$⊂**2** (500 MHz, D_2O, 298 K).

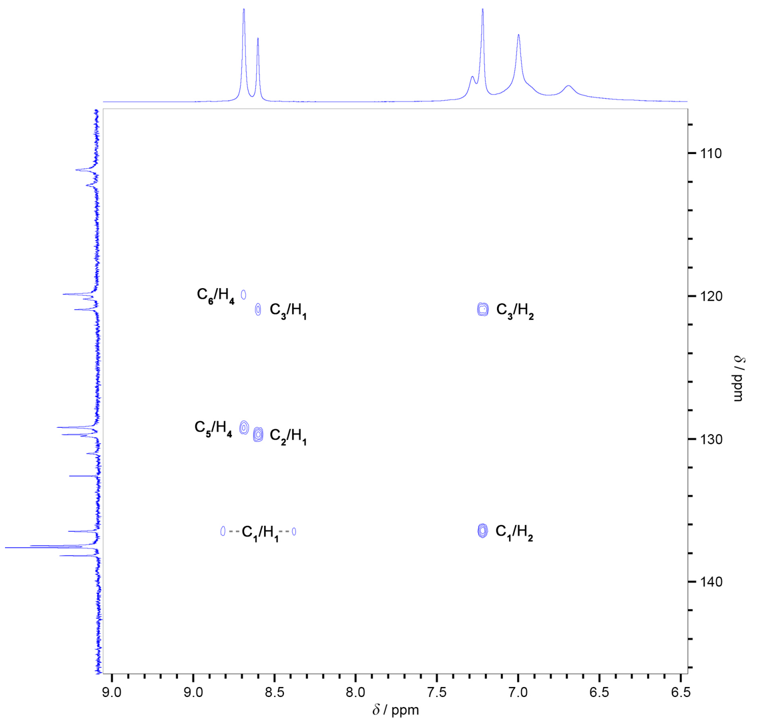

Figure S12: Partial ^1H-^{13}C HMBC NMR spectrum of $(E\text{-}\mathbf{1})_2\subset\mathbf{2}$ (500 MHz, D_2O, 298 K) (the corresponding full-range spectrum is shown in Figure S11).

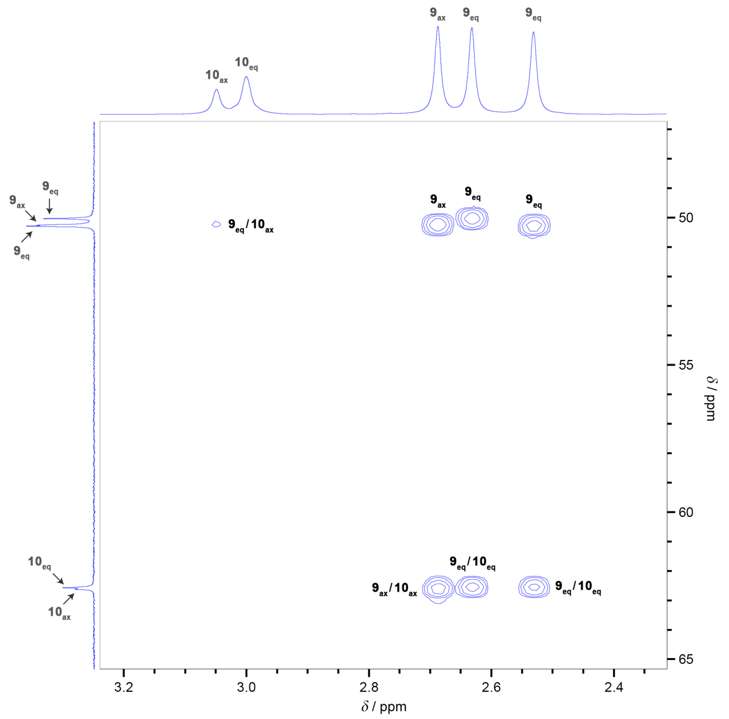

Figure S13: Partial ^1H-^{13}C HMBC NMR spectrum of $(E\text{-}\mathbf{1})_2\subset\mathbf{2}$ (500 MHz, D_2O, 298 K) (the corresponding full-range spectrum is shown in Figure S11).

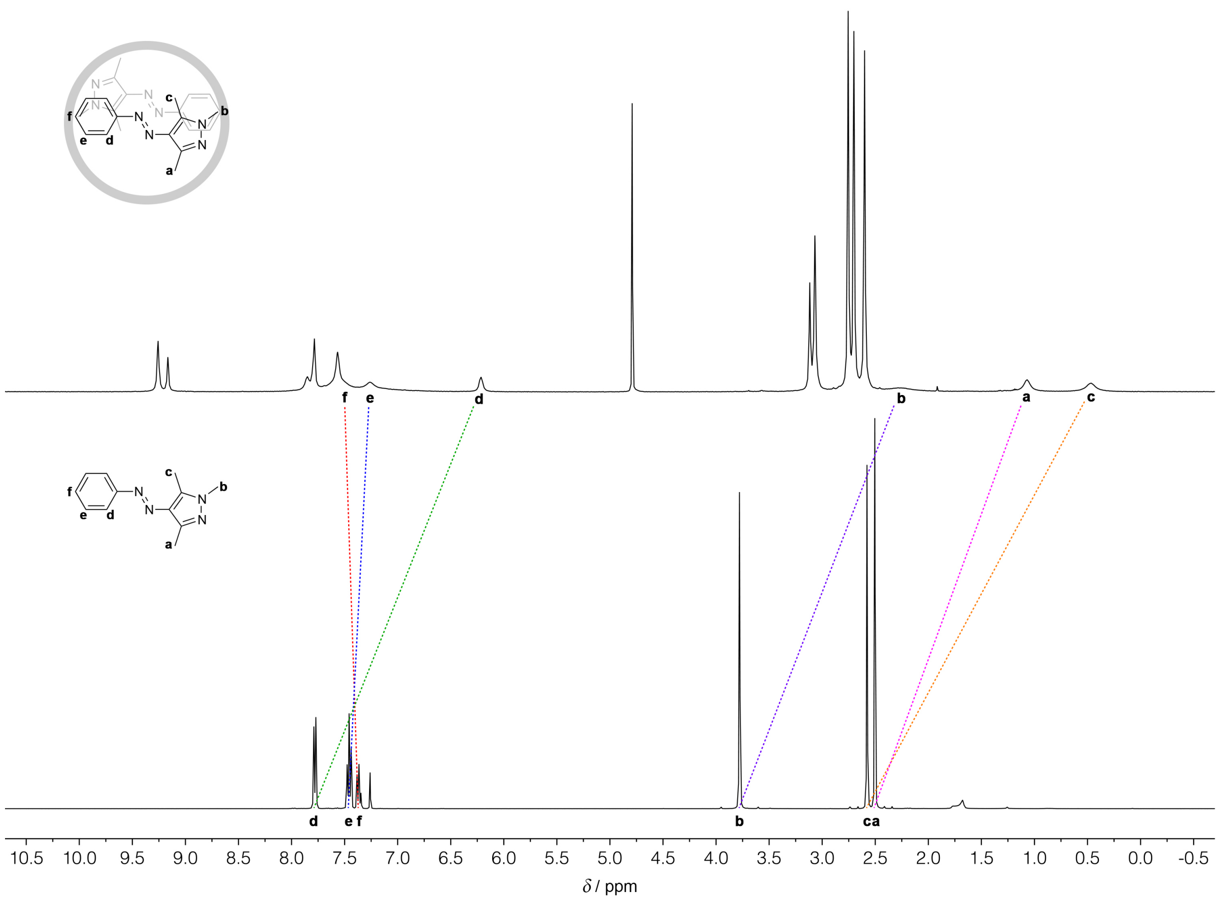

Figure S14: Encapsulation-induced shifts in the resonances of *E*-**1**'s protons. Bottom: ^{1}H NMR spectrum of *E*-**1** (500 MHz, $CDCl_3$, 298 K); top: ^{1}H NMR spectrum of $(E\text{-}\mathbf{1})_2{\subset}\mathbf{2}$ (500 MHz, D_2O, 298 K).

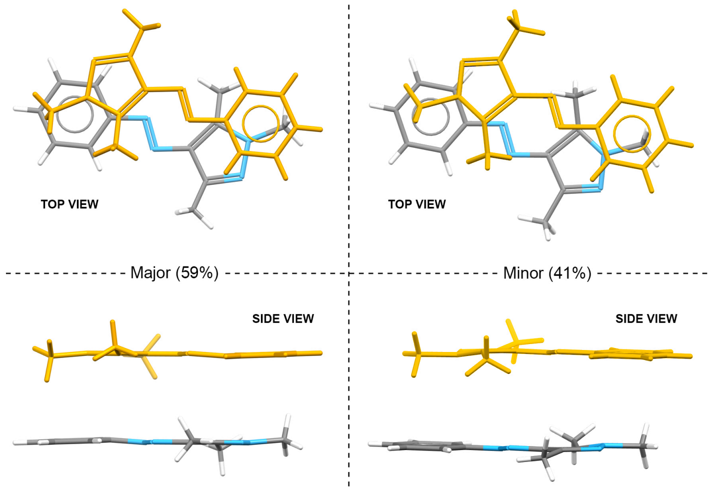

Figure S15: Comparison of the X-ray structures (wireframe representation) of the major (left) and the minor (right) conformations of *E*-**1** dimer within $(E\text{-}\mathbf{1})_2{\subset}\mathbf{2}$ in the solid state. Note the proximity of the *N*-bound methyl group of one guest molecule to the phenyl ring of the other molecule. One guest is shown in yellow, the other one – in gray, blue, and white (carbon, nitrogen, and hydrogen, respectively).

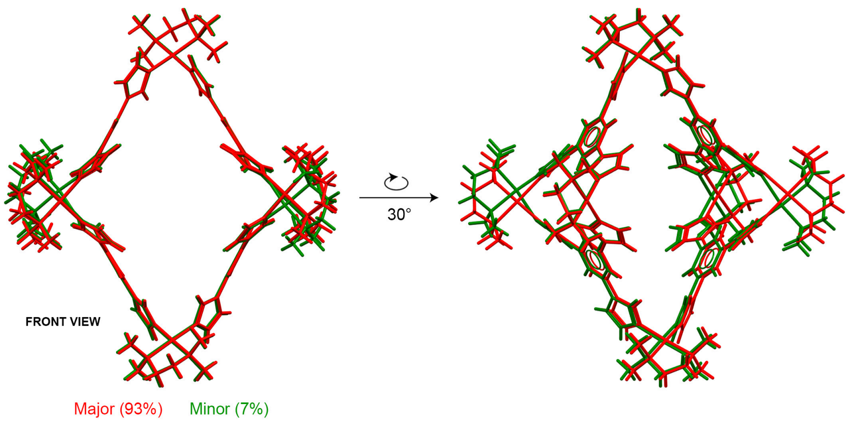

Figure S16: Comparison of the X-ray structures (wireframe representation) of the major (red) and minor (green) conformers within $(E\text{-}\mathbf{1})_2{\subset}\mathbf{2}$ in the solid state.

4. Reversible switching of $(E\text{-}\mathbf{1})_2\subset\mathbf{2}$

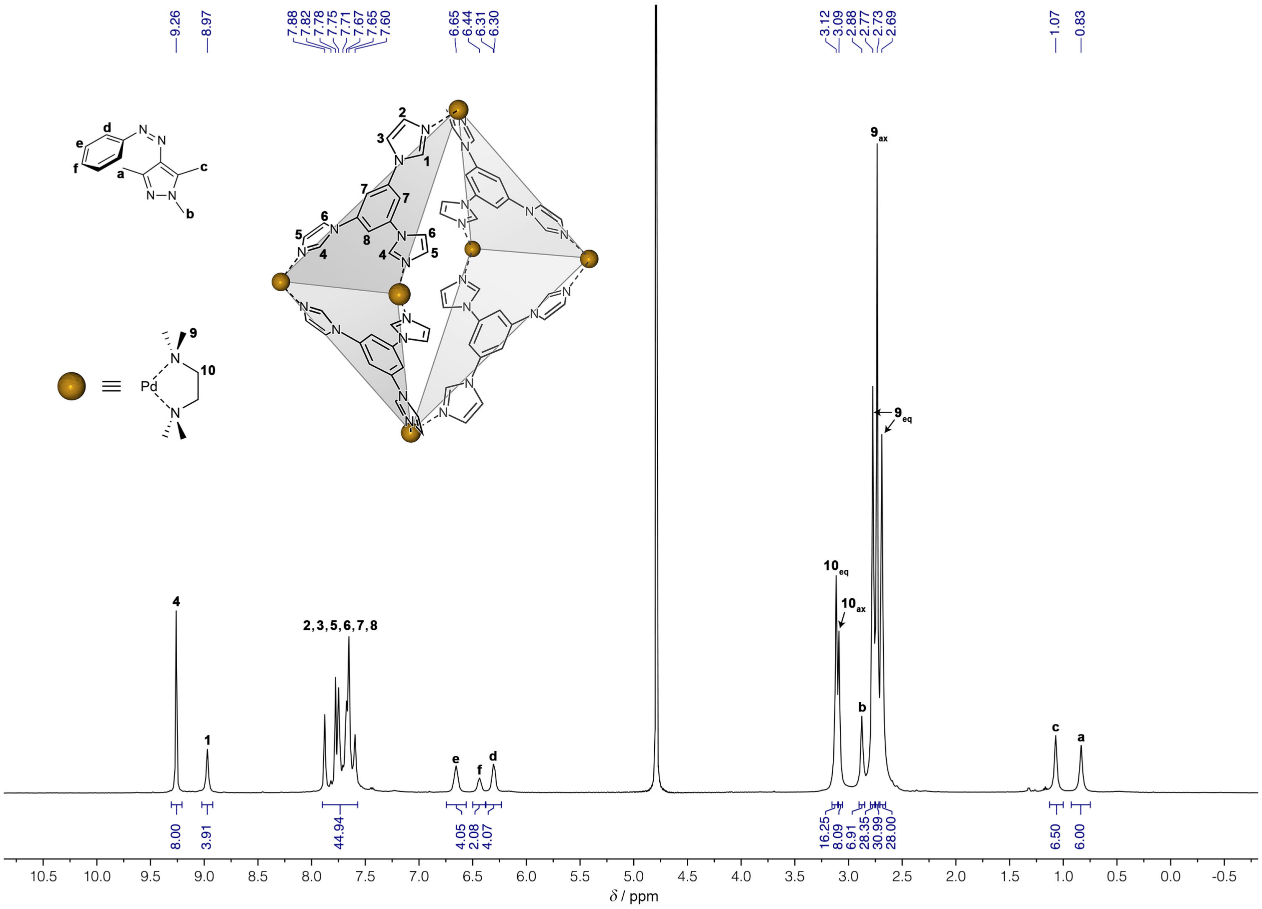

Figure S17: ^{1}H NMR spectrum of $(E\text{-}\mathbf{1})_2\subset\mathbf{2}$ exposed to UV light (i.e., a 1:1 mixture of $(Z\text{-}\mathbf{1})\subset\mathbf{2}$ and $Z\text{-}\mathbf{1}$) (500 MHz, D_2O, 298 K).

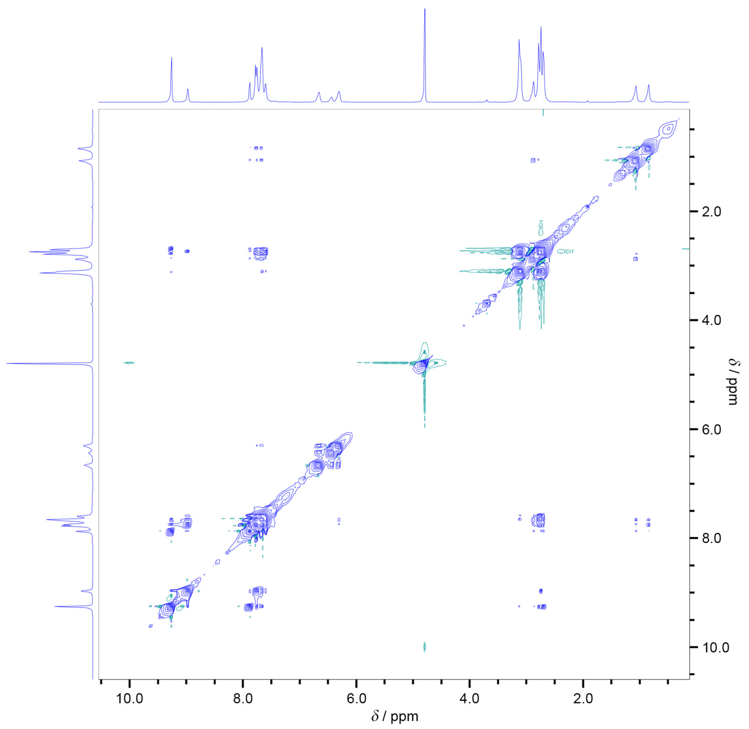

Figure S18: ^{1}H-^{1}H NOESY NMR spectrum of (*E*-**1**)$_2$⊂**2** exposed to UV light (i.e., a 1:1 mixture of (*Z*-**1**)⊂**2** and *Z*-**1** (500 MHz, D_2O, 298 K).

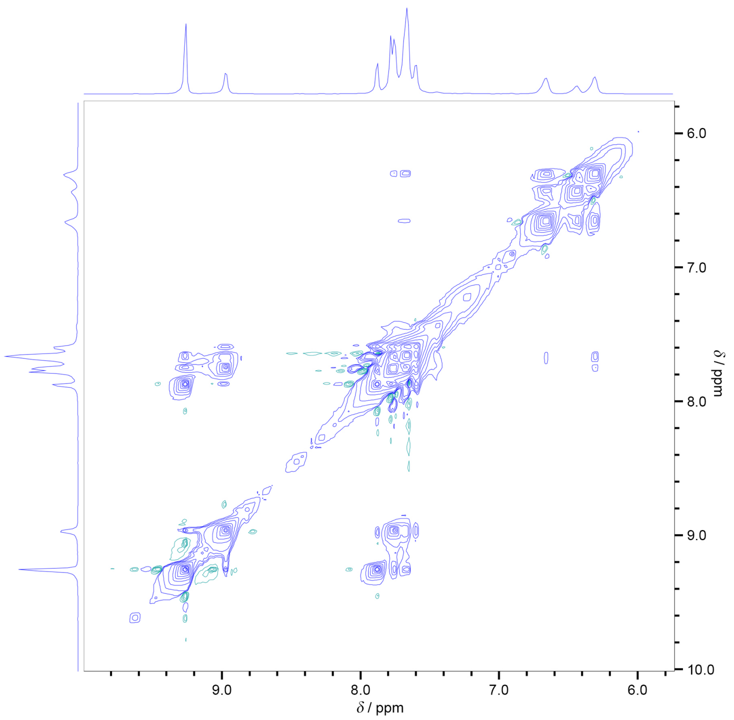

Figure S19: Partial ^{1}H-^{1}H NOESY NMR spectrum of (*E*-**1**)$_2$⊂**2** exposed to UV light (i.e., a 1:1 mixture of (*Z*-**1**)⊂**2** and *Z*-**1** (500 MHz, D_2O, 298 K) (the corresponding full-range spectrum is shown in Figure S18).

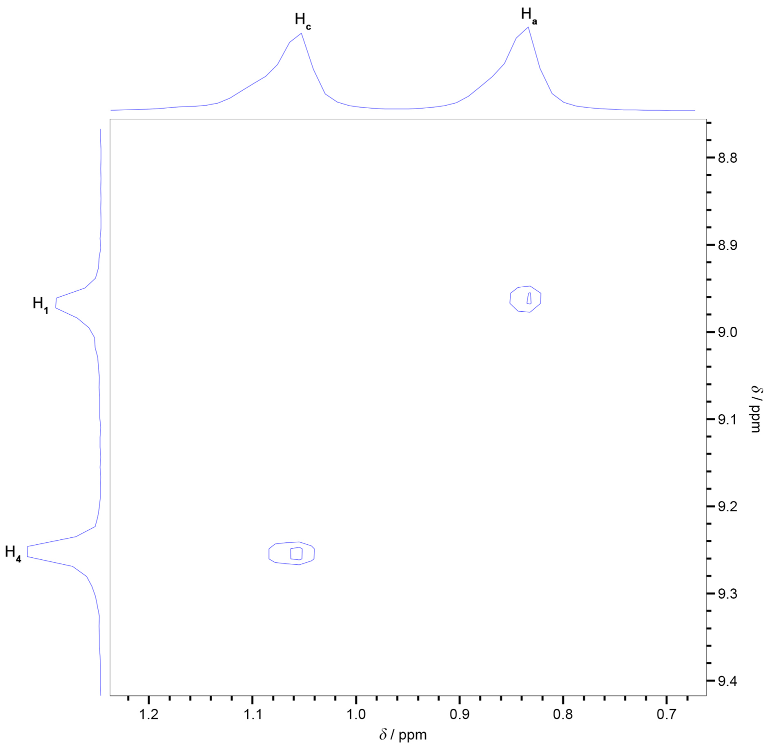

Figure S20: Partial ^{1}H-^{1}H NOESY NMR spectrum of (E-**1**)$_2$⊂**2** exposed to UV light (i.e., a 1:1 mixture of (Z-**1**)⊂**2** and Z-**1** (500 MHz, D_2O, 298 K) (the corresponding full-range spectrum is shown in Figure S18).

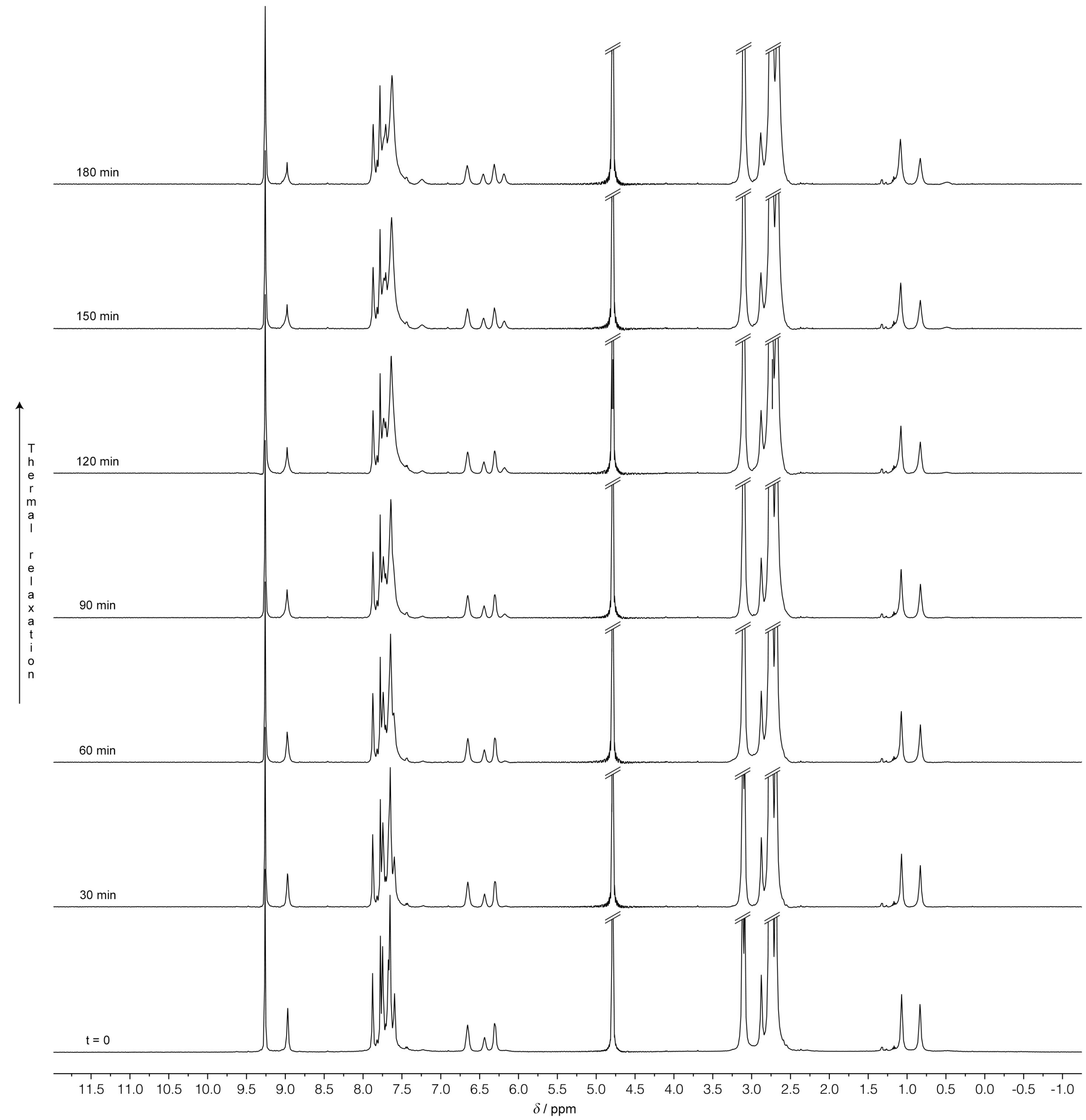

Figure S21: A series of ^{1}H NMR spectra (500 MHz, D_2O, 298 K) of (*E*-**1**)$_2$⊂**2** pre-irradiated with UV light (see Figure 6 in the main text) (i.e., (*Z*-**1**)⊂**2** + (*Z*-**1**)) before (bottom) and after thermal relaxation in the dark for different time periods up to 3 h.

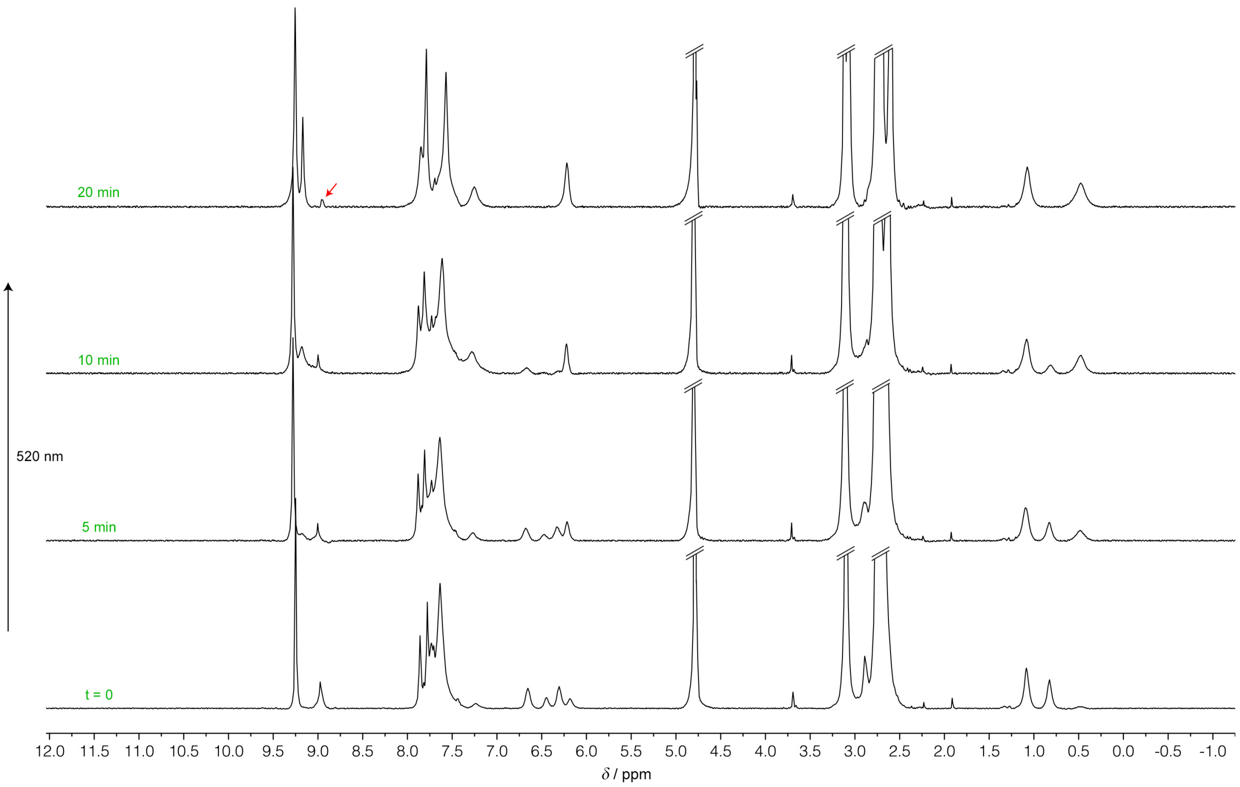

Figure S22: A series of ^{1}H NMR spectra (500 MHz, D_2O, 298 K) of (*E*-**1**)$_2$⊂**2** following irradiation with UV light (365 nm) for 25 min and thermal relaxation for 3 h before (bottom) and after exposure to green light (520 nm) inside the NMR spectrometer (using an optical fiber) for different time periods (denoted in green font) for up to 20 min. The red arrow in the final spectrum indicates residual **2** arising from (*Z*-**1**)⊂**2**.

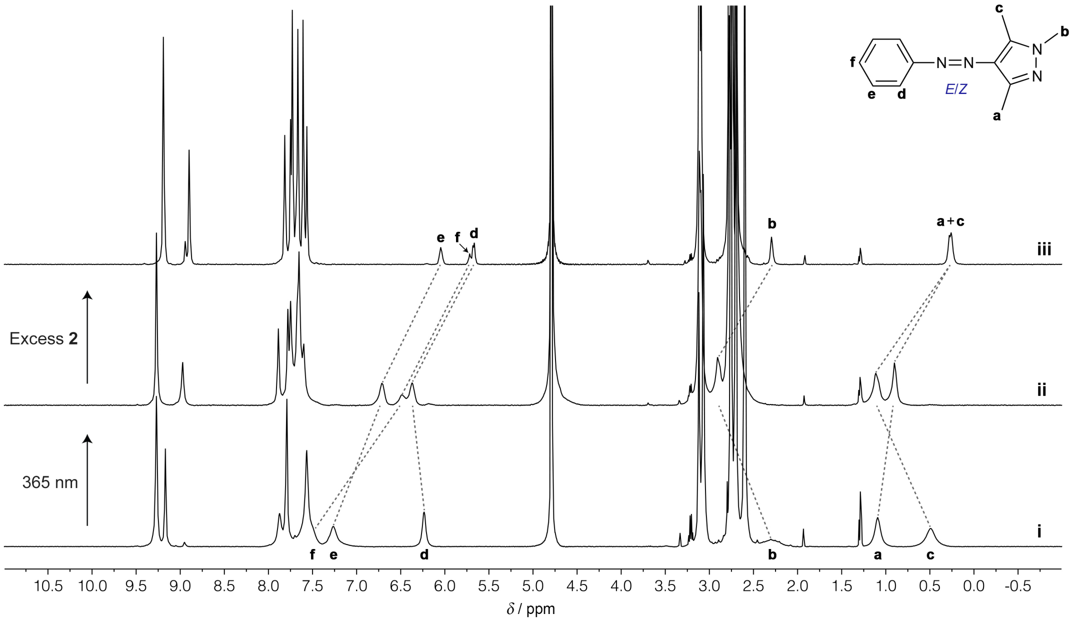

Figure S23: Shifts in the proton resonances of encapsulated **1** induced by UV light (causing expulsion of 50% of the guests) followed by the addition of an excess (extra 2.5 eq) of cage **2** (causing re-encapsulation). The small signals at 8.94, 1.92, and 1.29 ppm correspond to contaminations (500 MHz, D_2O, 298 K).

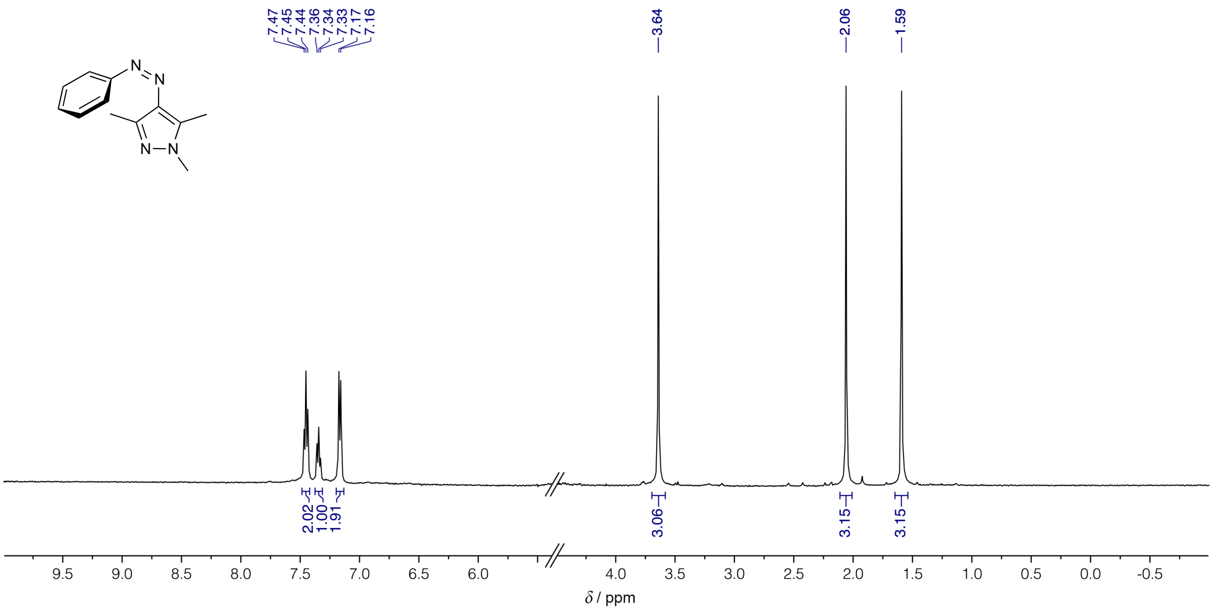

Figure S24: ^{1}H NMR spectrum of *Z*-**1** in water (500 MHz, D_2O, 298 K).

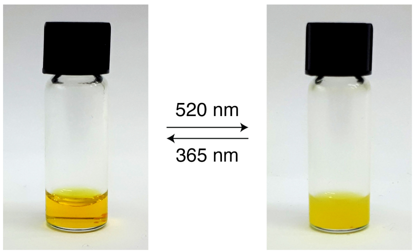

Figure S25: Photograph of a clear solution of *Z*-**1** in water (left) and a suspension obtained by exposure to green light (*E*-**1**; right).

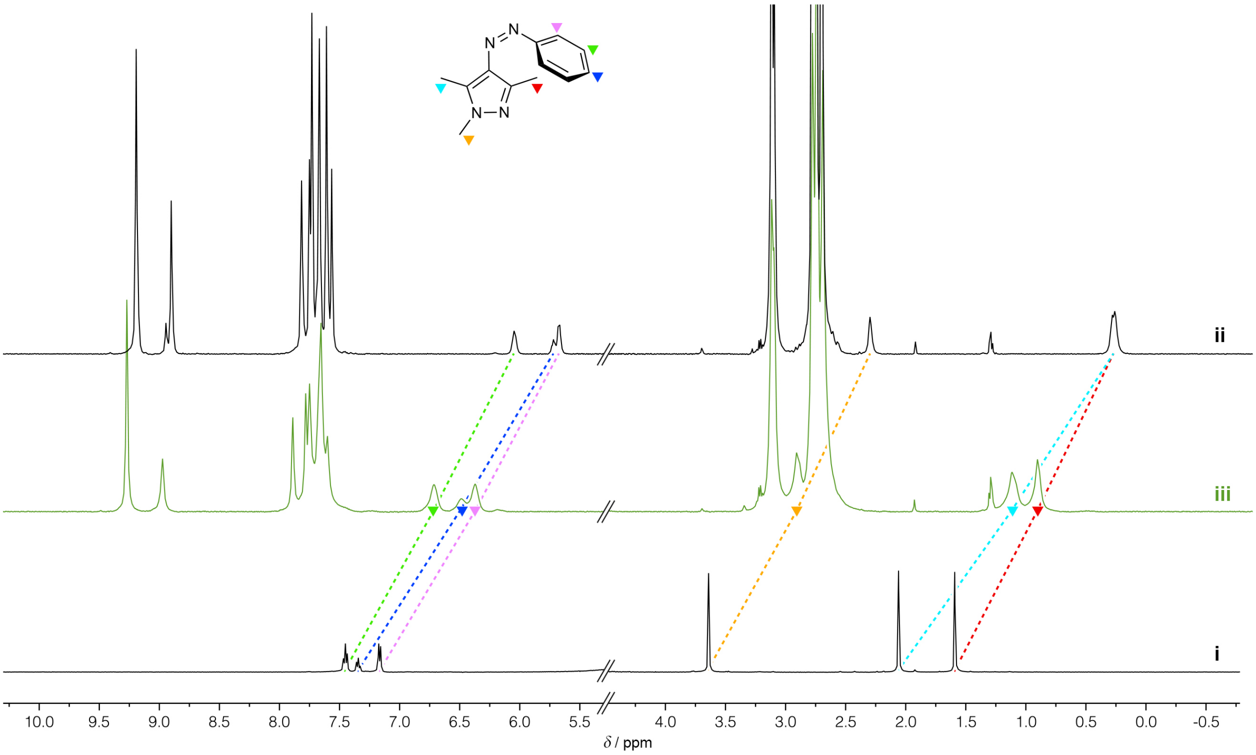

Figure S26: ^{1}H NMR spectra of *Z*-**1** (**i**; replotted from Figure S24), (*Z*-**1**)⊂**2** (**ii**; replotted from Figure S23), and a 1:1 mixture of *Z*-**1** and (*Z*-**1**)⊂**2** (**iii**; replotted from Figure S23).

5. X-ray data collection and structure refinement

Single crystals of inclusion complex $(E\text{-}\mathbf{1})_2\subset\mathbf{2}$ were obtained by slow evaporation of water from the aqueous solution. A data set was collected after immersion of a crystal in Paratone oil, followed by immediately flash-cooling under a liquid nitrogen stream. Single-crystal X-ray data were collected on a sealed-tube Rigaku XtaLAB^{PRO} dual source diffractometer equipped with a Pilatus 200K detector using Cu-Kα radiation (1.54184 Å). Data were collected under liquid nitrogen at 100 K. The data were processed with CrysAlis^{PRO} and the structures were solved by direct methods using SHELXT [2]. Refinement was carried out based on F^2 with SHELXL [3] with full matrix least-squares. Hydrogen atoms were assigned as isotropic in riding mode. *PLATON* SQUEEZE [4] was applied, eliminating the contribution of disordered water molecules. Crystallographic data and refinement parameters are summarized in Table S1.

CCDC No.	1939530
Formula	$C_{118.85}H_{170.66}N_{51.31}O_{23.09}Pd_6$
Formula weight	3326.31
Crystal system	Monoclinic
Space group	$P2_1/c$
Crystal size (mm)	0.178 × 0.103 × 0.082
Crystal color and shape	Yellow chunk
Temperature (K)	100
a (Å)	22.2791(5)
b (Å)	41.4992(4)
c (Å)	15.0897(3)
α (°)	90
β (°)	138.302(4)
γ (°)	90
Volume ($Å^3$)	9280.5(6)
Z	2
ρ_{calcd} (g cm^{-1})	1.190
μ (mm^{-1})	5.127
No. of reflections (unique)	177889 (15815)
R_{int}	0.0530
Completeness to θ (%)	99.9
Data / restraints / parameters	15815 / 225 / 990
Goodness-of-fit on F^2	1.024
Final R_1 and wR_2 indices [$I > 2\sigma(I)$]	0.0826, 0.2441
R_1 and wR_2 indices (all data)	0.0878, 0.2508

Table S1. Crystallographic data for (*E*-**1**)$_2$⊂**2**.

6. DFT calculations

Geometry optimizations were carried out within unconstrained C_1 symmetry *in vacuo* using Gaussian software [5]. The starting coordinates of the (*Z*-**1**)⊂**2** complex were derived from the X-ray structure of the 1:2 complex of **2** and tetra-*o*-fluoroazobenzene [6], modified to account for the NOE correlations observed between *Z*-**1** and **2** (Figures S18–S20). The model was optimized as a dication with ten NO_3^- anions, whose positions were based on the crystal structure of the cage [6] (the remaining two NO_3^- anions were omitted to avoid arbitrariness; optimization without any counterions included led to unrealistic results). The calculations were performed at the B3LYP/6-31G(d,p)/LANL2DZ(Pd) level of theory [7, 8] and an empirical dispersion correction D3 [9].

7. Supporting references

[1] Weston, C. E.; Richardson, R. D.; Haycock, P. R.; White, A. J. P.; Fuchter, M. J. *J. Am. Chem. Soc.* **2014**, *136*, 11878–11881. doi:10.1021/ja505444d

[2] Sheldrick, G. M. *Acta Crystallogr. A* **2015**, *71*, 3–8. doi:10.1107/s2053273314026370

[3] Sheldrick, G. M. *Acta Crystallogr. C* **2015**, *71*, 3–8. doi:10.1107/S2053229614024218

[4] Spek, A. L. *Acta Crystallogr. C* **2015**, *71*, 9–18. doi:10.1107/S2053229614024929

[5] Frisch, M. J.; Trucks, G. W.; Schlegel, H. B.; Scuseria, G. E.; Robb, M. A.; Cheeseman, J. R.; Scalmani, G.; Barone, V.; Petersson, G. A.; Nakatsuji, H.; Li, X.; Caricato, M.; Marenich, A. V.; Bloino, J.; Janesko, B. G.; Gomperts, R.; Mennucci, B.; Hratchian, H. P.; Ortiz, J. V.; Izmaylov, A. F.; Sonnenberg, J. L.; Williams-Young, D.; Ding, F.; Lipparini, F.; Egidi, F.; Goings, J.; Peng, B.; Petrone, A.; Henderson, T.; Ranasinghe, D.; Zakrzewski, V. G.; Gao, J.; Rega, N.; Zheng, G.; Liang, W.; Hada, M.; Ehara, M.; Toyota, K.; Fukuda, R.; Hasegawa, J.; Ishida, M.; Nakajima, T.; Honda, Y.; Kitao, O.; Nakai, H.; Vreven, T.; Throssell, K.; Montgomery, J. A., Jr.; Peralta, J. E.; Ogliaro, F.; Bearpark, M. J.; Heyd, J. J.; Brothers, E. N.; Kudin, K. N.; Staroverov, V. N.; Keith, T. A.; Kobayashi, R.; Normand, J.; Raghavachari, K.; Rendell, A. P.; Burant, J. C.; Iyengar, S. S.; Tomasi, J.; Cossi, M.; Millam, J. M.; Klene, M.; Adamo, C.; Cammi, R.; Ochterski, J. W.; Martin, R. L.; Morokuma, K.; Farkas, O.; Foresman, J. B.; Fox, D. J. Gaussian 16, Revision B.01. Gaussian, Inc., Wallingford CT, 2016.

[6] Samanta, D.; Gemen, J.; Chu, Z.; Diskin-Posner, Y.; Shimon, L. J. W.; Klajn, R. *Proc. Natl. Acad. Sci. USA* **2018**, *115*, 9379–9384. doi:10.1073/pnas.1712787115

[7] Lee, C.; Yang, W.; Parr, R. G. *Phys. Rev. B* **1988**, *37*, 785–789. doi:10.1103/PhysRevB.37.785

[8] Becke, A. D. *J. Chem. Phys.* **1993**, *98*, 5648–5652. doi: 10.1063/1.464913

[9] Grimme, S.; Antony, J.; Ehrlich, S.; Krieg, H. *J. Chem. Phys.* **2010**, 132, 154104. doi: 10.1063/1.3382344
